# Combination therapies for chronic hepatitis B in the era of emerging novel drugs

**DOI:** 10.1007/s12072-025-10888-2

**Published:** 2025-10-17

**Authors:** Dandan Weng, Chenxi Zhang, Qunyan Wei, Lukan Zhang, Xinya Zang, Guancheng Huang, Zhujun Cao, Qing Xie

**Affiliations:** 1 Department of Infectious Diseases, Yangming Hospital Affiliated to Ningbo University, No. 800, Chengdong Road, Yuyao, 315400 Zhejiang China; 2 Department of Infectious Diseases, Ruijin Hospital, Shanghai Jiao Tong University School of Medicine, No. 197, Ruijin 2nd Road, Huangpu District, Shanghai, 200025 China; 3 Department of Hepatology, Qiannan People’s Hospital, No. 9 Wenfeng Road, Duyun City, 558000 Qiannan Buyi and Miao Autonomous Prefecture, Guizhou Province China

**Keywords:** Hepatitis B virus (HBV), Antiviral treatment, Functional cure, Treatment strategies, Immune response, Personalized medicine, Multidrug therapy, Novel antiviral agents, HBsAg clearance, Personalized treatment

## Abstract

**Background/objectives:**

Chronic Hepatitis B (CHB) is a global health concern, affecting hundreds of millions and potentially leading to severe outcomes, such as cirrhosis and hepatocellular carcinoma. The primary treatment goal is to achieve a functional cure—defined as the loss of hepatitis B surface antigen (HBsAg) 24 weeks after the cessation of therapy—which reduces liver inflammation, improves histopathology, and decreases the incidence of end-stage liver disease. However, this goal is rarely achieved with current therapies, especially monotherapies. With a deeper understanding of the HBV lifecycle and its interactions with the host immune system, combination therapy strategies are increasingly demonstrating potential to enhance treatment outcomes for CHB.

**Methods:**

This article reviews the application of novel drugs in combination therapy, analyzes the suitability of different drug combinations, and evaluates their effects on HBsAg clearance rates and overall cure rates.

**Results:**

The review identified several promising drugs, such as capsid assembly modulators, entry inhibitors, and RNA interference therapies, which demonstrated greater efficacy in combination therapy, achieving higher HBsAg clearance and enhanced immune responses compared to monotherapies. However, effectiveness varied among patient subgroups, highlighting the need for personalized treatment.

**Conclusions:**

Combination therapies involving novel drugs hold promise for improving CHB outcomes, particularly in achieving a functional cure. Further research is required to optimize personalized regimens and to assess their long-term safety and efficacy for broader clinical use.

**Supplementary Information:**

The online version supplementary material available at 10.1007/s12072-025-10888-2.

## Introduction

Chronic Hepatitis B (CHB) is a major global health concern, with an estimated 254 million affected worldwide [1]. Patients with CHB are at risk of developing cirrhosis, hepatocellular carcinoma (HCC), and related complications, which contributes to approximately 1.1 million deaths annually [2].

Current antiviral therapies effectively suppress the virus but fail to achieve a functional cure, leaving the risk of liver fibrosis and HCC progression.

**Fig. 1:**
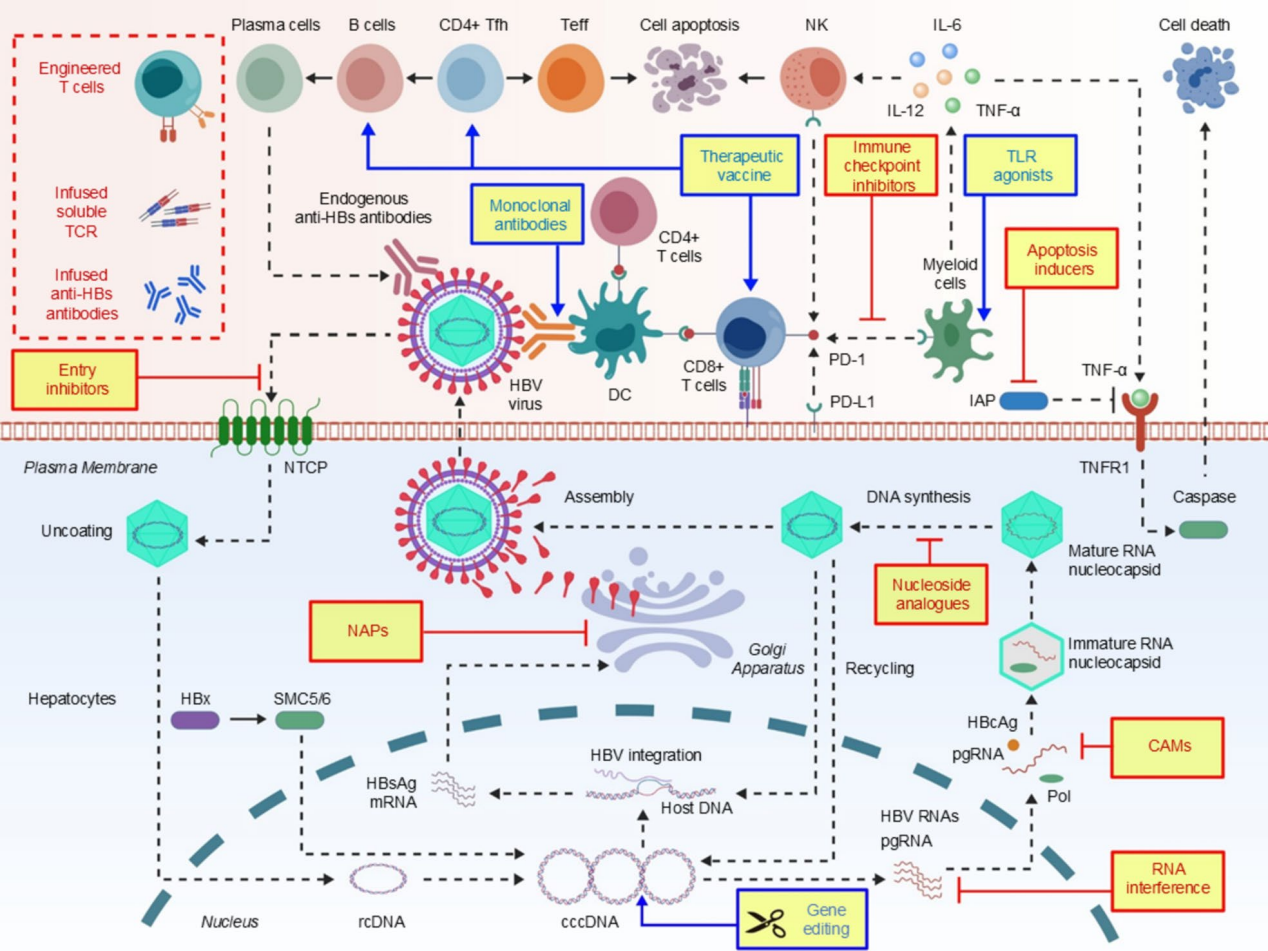
Hepatitis B virus (HBV) life cycle and immuno-antiviral intervention strategies. HBV infects hepatocytes by binding to sodium taurocholate co-transporting polypeptide (NTCP), facilitating endocytic entry. After uncoating, relaxed circular DNA (rcDNA) is transported to the nucleus and converted by host enzymes into covalently closed circular DNA (cccDNA), which serves as the template for pregenomic RNA (pgRNA) and messenger RNAs (mRNAs) encoding viral proteins. The HBV X protein (HBx) promotes cccDNA transcription by degrading the host restriction factor structural maintenance of chromosomes complex 5/6 (SMC5/6). In the cytoplasm, pgRNA and viral polymerase (Pol) are encapsidated into nucleocapsids, where reverse transcription produces rcDNA. Mature capsids may be enveloped and secreted as complete virions (Dane particles) or released in enveloped or non-enveloped forms via the endoplasmic reticulum–Golgi–multivesicular body (ER–Golgi–MVB) pathway. Some capsids return to the nucleus to replenish the cccDNA pool, while others integrate into the host genome. Hepatitis B surface antigen (HBsAg)-only subviral particles (SVPs) are abundantly secreted to aid immune evasion. Drug targets include: (1) viral entry inhibitors, (2) cccDNA-targeting approaches (e.g., gene editing, epigenetic silencing), (3) RNA interference against pgRNA and viral mRNAs, (4) core assembly modulators (CAMs), (5) HBV polymerase inhibitors (nucleos(t)ide analogs), (6) HBsAg secretion blockers (e.g., nucleic acid polymers [NAPs]), and (7) immunotherapies including therapeutic vaccines, immune checkpoint inhibitors, toll-like receptor (TLR) agonists, monoclonal antibodies, and gene-engineered T cells

Recent advancements in the understanding of the HBV life cycle and its interplay with the host immune system have underscored the efficacy of combination therapy approaches [3, 4]. Figure 1 illustrates the HBV life cycle and highlights how targeting multiple steps can enhance viral suppression, restore immune responses, and reduce antigen levels—offering a promising strategy to address current treatment limitations.

This review summarizes recent advances in novel antiviral and immune-based therapies for CHB, with a focus on combination strategies aiming for functional cure. Clinical trials discussed were selected based on Phase II or later status and/or the inclusion of functional cure–related endpoints. While these therapies are not yet included in current guidelines, they align with the strategic goals emphasized by both the European Association for the Study of the Liver (EASL) and the American Association for the Study of Liver Diseases (AASLD) to advance toward finite, curative treatments.

## Chronic HBV infection, standard treatment and functional cure

CHB persists due to cccDNA formation and viral DNA integration, both maintaining HBsAg production [5]. Current therapies—nucleos(t)ide analogs (NAs) and pegylated interferon-α (Peg-IFNα)—suppress replication and modulate immunity but fail to eliminate cccDNA or block HBsAg expression. Functional cure, defined as sustained HBsAg loss after treatment, remains rare [6]. For more details, see Appendix 1.

## Novel drugs in combination therapy

**Fig. 2:**
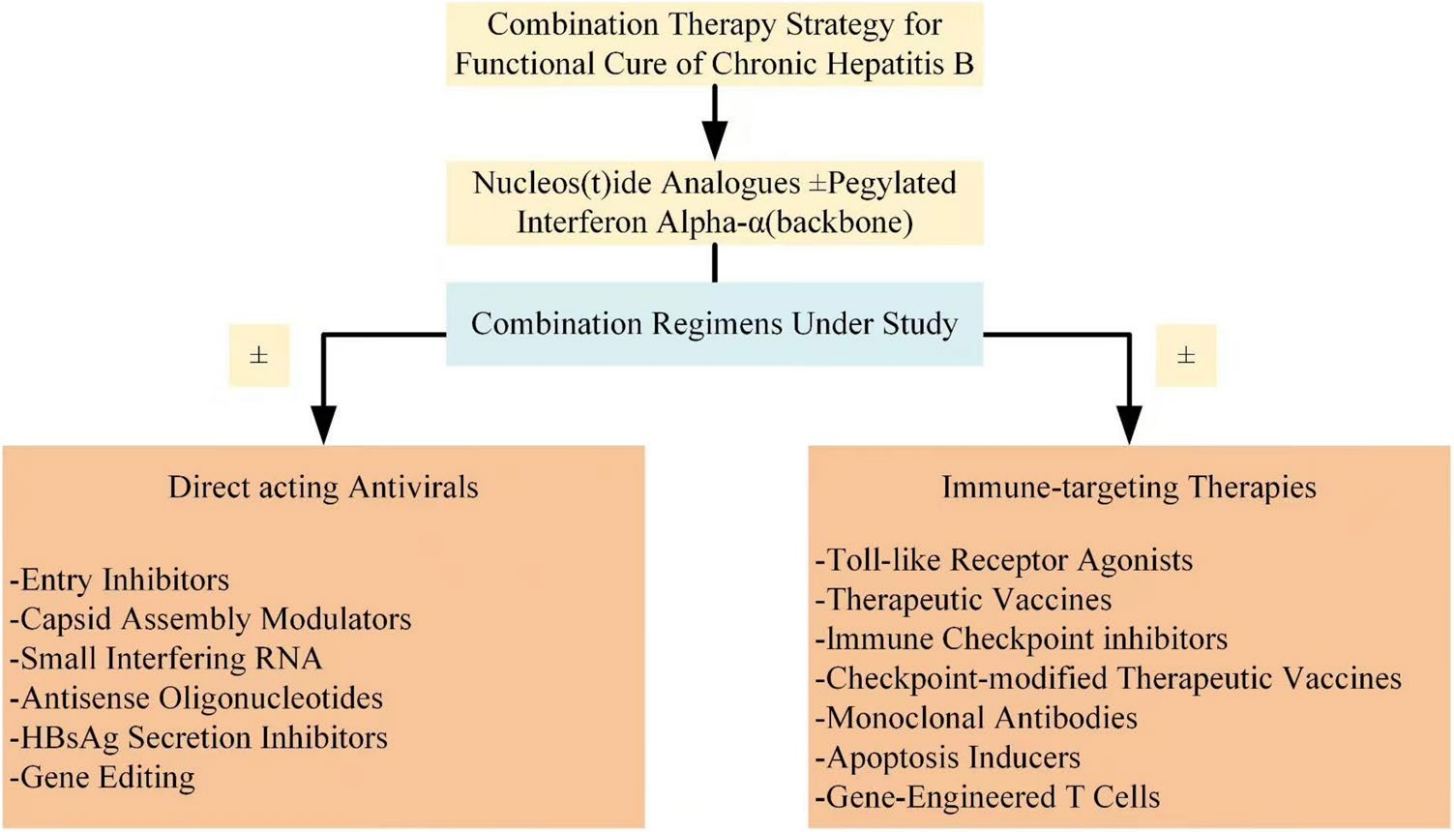
Flowchart of combination strategies toward functional cure of chronic hepatitis B

Recent drug development has expanded combination therapy options, including direct antivirals targeting the HBV life cycle (Table S1 in the Appendix S2) and immune modulators restoring HBV-specific immunity or overcoming immune blockade (Table S2 in the Appendix S2). Most are combined with NAs or Peg-IFNα. Fig. 2 summarizes these strategies by drug class and therapeutic goal.

### Direct-acting antivirals

#### Entry inhibitors

Entry inhibitors target NTCP, a key receptor on hepatocytes essential for HBV and HDV entry. Bulevirtide, a myristoylated peptide from the HBV preS1 domain, binds NTCP to block viral entry [7].

As monotherapy, bulevirtide shows limited efficacy in reducing HBsAg. In a phase III trial in chronic HBV/ HDV co-infection, no HBsAg loss or ≥ 1 log₁₀ IU/mL decline was observed after 48 weeks, though 20% of patients in the 10 mg group achieved undetectable HDV RNA [7,8]. In contrast, bulevirtide combined with Peg-IFNα led to more pronounced HBsAg reductions. At the end of treatment (EOT), ≥ 1 log₁₀ IU/mL HBsAg reduction occurred in 16% and 12% of patients in the 2 mg and 10 mg combination groups, respectively. HBsAg loss was observed in 8% and 4% after EOT (W24), and 10% and 4% at W48, respectively. HDV RNA suppression was also enhanced. At W24, undetectable HDV RNA was achieved in 17% with Peg-IFNα alone, 32% and 46% with the 2 mg and 10 mg combinations, and 12% with bulevirtide monotherapy. At W48, HDV RNA remained undetectable in 26% and 46% in the respective combination groups [9]. Notably, most patients with undetectable HDV RNA did not achieve HBsAg loss. Overall, the 2 mg combination achieved higher HBsAg loss rates, while the 10 mg group showed greater HDV RNA suppression.

Combination therapy with higher doses and longer duration improves outcomes, especially in patients with lower baseline HDV RNA and compensated liver function. Bulevirtide received European Medicines Agency (EMA) approval in 2020 for chronic hepatitis D.

#### Capsid assembly modulators (CAMs)

Capsid assembly modulators (CAMs) inhibit HBV replication by disrupting capsid formation and pgRNA encapsidation, also impeding cccDNA amplification. CAMs are classified as Class I (aberrant capsid assembly) or Class II (empty capsid formation) [10].

GLS4, a Class I CAM-A and the first to reach phase III trials, has limited monotherapy potential due to poor pharmacokinetics, but co-administration with ritonavir (RTV) enhances exposure and antiviral efficacy by inhibiting hepatic metabolism [11]. Initial clinical trials suggest that CAMs alone do not surpass NAs in effectiveness. In a phase 2b trial, GLS4/RTV combined with ETV demonstrated greater antiviral activity than ETV monotherapy in both treatment-naïve and virally suppressed patients with chronic hepatitis B [12]. The main results are presented in Table 1, with further details provided in the Appendix S3.

Bersacapavir (JNJ-6379), a representative Class II, was evaluated in the REEF-1 study in combination with NAs, with or without the siRNA agent JNJ-3989 [13]. Although this regimen demonstrated modest antiviral activity, its effect on HBsAg levels was limited. The main results are presented in Table 1, with further details provided in the Appendix S3.

Other CAMs in development, such as ABI-H3733, ALG-000184, and EDP-514, show promising antiviral activity. ALG-000184, with dual Class I/II properties, has demonstrated additive effects in combination with NAs, supporting its potential role in cure-focused regimens (see Table 1).

**Table 1:** Combination approaches targeting CHB functional cure

Drug Class	Representative Agent and Combined Protocols	EOT Key Events (HBV DNA ↓, HBsAg ↓/Clearance)	Functional Cure (HBsAg Loss, 24w Off-Tx)
Entry Inhibitor	**Bulevirtide** ± Peg-IFNα	HBsAg ↓ ≥ 1 log in 16% (2 mg) and 12% (10 mg) at EOT	HBsAg loss: 8%(2 mg); 4%(10 mg); none with monotherapy
CAM-A	**GLS4** + Ritonavir + Entecavir	Treatment-naïve: HBV DNA and HBsAg LSM reduction at W48: − 6.28/ − 0.87 vs − 5.72/ − 0.65 log₁₀ IU/mL (combo vs mono); Virally suppressed: pgRNA and HBsAg LSM reduction: − 1.61/ − 0.17 vs − 0.28/ − 0.06 log₁₀ IU/mL; none HBsAg loss	not reported
CAM-E	**JNJ-6379** + NA ± siRNA(JNJ-3989)	JNJ-3989 + NA (dual): HBV DNA ↓ (marked, dose-dependent); HBsAg ↓ –1.5 to –2.6 log; HBsAg loss 1–3% / 0% seroconv JNJ-6379 + NA (dual): HBV DNA ↓ (mild); HBsAg ↓ –0.07 log; HBsAg loss 0% JNJ-3989 + JNJ-6379 + NA (triple): HBV DNA ↓ (dose-dependent, sustained post-treatment); HBsAg ↓ –1.8 log; HBsAg loss 0% at EOT	JNJ-3989 + NA (dual): HBsAg ↓ –1.0 to –1.9 log (24W off-Tx); 3 pts cleared HBsAg; 1 pt met full functional cure (off NA); others remained on NA, HBeAg/HBcrAg + JNJ-6379 + NA (dual): HBsAg ↓ –0.15 log (24W off-Tx); No HBsAg loss; No functional cure JNJ-3989 + JNJ-6379 + NA (triple): HBsAg ↓ –1.4 log (24W off-Tx); No HBsAg loss; No functional cure
CAM (Class II / Hybrid)	ALG-000184 (monotherapy or + entecavir)	HBV DNA ↓ up to 4.2 log₁₀; HBsAg ↓ up to 0.8 log₁₀ with monotherapy; up to 1.65 log₁₀ with combo	Not reported;
siRNA	Xalnesiran + Peg-IFNα-2a + NA	HBsAg loss/seroconversion at EOT: 3–7% / 3% (mono); 30% / 23% (como); HBV DNA < 10 IU/mL in most patients	HBsAg loss / seroconversion: 3–7% / 0 ~ 3% (mono); 23% / 23% (como);HBV DNA < 10 IU/mL in most patients
siRNA	**VIR-2218** + Peg-IFNα	HBsAg ↓ up to –3.0 log₁₀; loss in 28–31%,no HBV DNA rebound	HBsAg loss 持续28–31%,; anti-HBs in 91% of seroclearers
ASO	**Bepirovirsen** + NA ± PEG-IFNα	Bepirovirsen ± NA (B-Clear Study, 300 mg × 24w) EOT: HBsAg < LLOD + HBV DNA < LLOQ → 26% (On-NA), 29% (Not-on-NA) Bepirovirsen → PEG-IFNα (B-Together Study, NA-experienced) EOT: HBsAg loss + HBV DNA undetectable → • 22% (BPV 24w → PEG-IFNα 24w) • 17% (BPV 12w → PEG-IFNα 24w)	Bepirovirsen ± NA (B-Clear Study, 300 mg × 24w) At 24w Off-Tx: HBsAg < LLOD + HBV DNA < LLOQ in ~ 9–10% Bepirovirsen → PEG-IFNα (B-Together Study, NA-experienced) At 24w Off-Tx: HBsAg loss + HBV DNA undetectable: • 9% (BPV 24w → PEG-IFNα 24w) • 15% (BPV 12w → PEG-IFNα 24w) Earlier PEG-IFNα switch associated with lower relapse rates
HBsAg secretion inhibitors	**REP 2139/2165** + TDF + Peg-IFNα	60% achieved HBsAg ≤ 0.05 IU/mL; significant HBsAg reduction and seroconversion	14 (35%) achieved HBsAg seroconversion; 13 (32.5%) maintained virologic control at 24w off-Tx
Gene Editing	CRISPR/Cas9 + RNAi/NA	HBV DNA↓, HBsAg↓(20–30%), cccDNA↓(up to 70%); HBsAg undetectable in mice (RNAi), DHBV DNA↓97–98% (Entecavir), cccDNA clearance Cas9-driven	Not yet demonstrated in humans
Gene Editing	PBGENE-HBV	HBsAg ↓ ≥ 0.3 log₁₀ in 2/3 patients at 4 weeks; preclinical cccDNA ↓ 0.5 log₁₀	Not yet achieved; functional cure under investigation
TLR-7 Agonists	Ruzotolimod + Xalnesiran	18% achieved HBsAg seroclearance at EOT	12% maintained HBsAg loss at 24 weeks post-treatment
TLR-8 Agonists	GS-9688 ± TAF	Did not achieve ≥ 1 log HBsAg ↓; no significant HBsAg clearance	Not achieved
Protein-based vaccines	VBI-2601 + VIR-2218	HBsAg ↓ 1.7–1.8 log IU/mL; Anti-HBs seroconversion in > 30%	Not achieved; under evaluation
Epitope peptide-based vaccines	GS-4774 + Tenofovir	No significant HBsAg ↓; strong CD8 + T-cell cytokine response (↑ IFN-γ, TNF, IL-2)	Not observed
DNA-based vaccines	VTP-300 + Nivolumab / AB-729	29% had > 1 log HBsAg ↓ with VTP-300 + nivolumab; 97% maintained HBsAg < 100 IU/mL post AB-729	Infrequent; under study
Checkpoint-modified vaccines	VRON 0200 + Elebsiran + Tobevibart	Rapid HBsAg ↓ (–3.6 to –1.3 log₁₀ IU/mL by Day 35); all patients < 10 IU/mL; universal HBsAb seroconversion	2/6 achieved HBsAg loss (< 0.05 IU/mL) by Day 140
ICI(PD-L1)	Envafolimab	Mean HBsAg ↓ 0.309 log; 43% (3/7) with baseline HBsAg < 100 IU/mL achieved HBsAg clearance	not reported
ICI(PD-1)	Nivolumab ± GS-4774	13.6% had > 0.5 log HBsAg ↓ by Week 24; 1 patient (4.5%) maintained HBsAg loss at 12 months	1/22 achieved sustained HBsAg loss
Monoclonal antibodies	VIR-3434 + VIR-2218 (± Peg-IFNα)	Single-dose VIR-3434: HBsAg ↓ ~ 1.8 log, HBV DNA ↓ ~ 2.0 log; Combo with VIR-2218: ~ 15% achieved HBsAg loss;	durability under evaluation
Apoptosis inducer	APG-1387 ± Entecavir	Monotherapy: HBV DNA ↓ 0.38 log₁₀ IU/mL (Day 28); Sequential combo: HBV DNA ↓ 4.69 log₁₀, HBsAg ↓ 1.06 log₁₀, HBeAg ↓ 1.73 log₁₀ (Day 112)	Not yet achieved; functional cure under evaluation
Gene-engineered T cells	SCG101 ± NAs	94% had 1.0–4.6 log₁₀ HBsAg reduction at 28 days; DNA already suppressed	23.5% achieved complete HBsAg clearance (up to 1 year)

**Abbreviations:** CAMs, Capsid Assembly Modulators; CAM-A, Class I CAM; CAM-E, Class II CAM; siRNA, Small Interfering RNA; ASO, Antisense Oligonucleotide; HBsAg, Hepatitis B Surface Antigen; HBV DNA, Hepatitis B Virus DNA; cccDNA, Covalently Closed Circular DNA; TLR, Toll-Like Receptor; ICI, Immune Checkpoint Inhibitor; PD-1, Programmed Cell Death Protein 1; PD-L1, Programmed Death-Ligand 1; EOT, End of Treatment; Tx, Treatment; w, week(s); ALT, Alanine Aminotransferase; NA(s), Nucleos(t)ide Analogues; IFN-α, Interferon-alpha; LLOQ, Lower Limit of Quantification

#### Small interfering RNA (siRNA)

siRNA-based therapies suppress HBV replication by degrading viral transcripts from cccDNA or integrated DNA [14,15]. In a phase 2 trial of virally suppressed patients on stable NA therapy, xalnesiran monotherapy led to limited HBsAg loss (3–7% at EOT), while combination regimens with ruzasvir or Peg-IFNα showed higher efficacy [16]. Combination therapies with VIR-2218 or JNJ-3989 also demonstrated improved results in HBsAg reduction [13,17]. For the main results, see Table 1; further details on mechanisms and experimental data are in the Appendix S3.

#### Antisense oligonucleotides (ASOs)

ASOs are synthetic, single-stranded oligonucleotides that possess unique biochemical characteristics. They attach to complementary HBV RNA transcripts, forming ASO-RNA hybrids that are subsequently cleaved by ribonuclease H [18]. Among the ASOs under development, bepirovirsen (IONIS-HBVRx, GSK 3228836) represents one of the most advanced. Results from the phase 2b B-Clear study indicate that 24 weeks post-treatment, 9% of subjects in the combination therapy cohort (NAs and bepirovirsen) and 10% in the bepirovirsen monotherapy cohort achieved the primary endpoint (HBsAg < 0.05 IU/mL and HBV DNA < 20 IU/mL), with the combination therapy group reporting fewer adverse events [19]. Moreover, the B-TOGETHER study demonstrated that in NA-experienced patients, 24 weeks of bepirovirsen treatment, or 12 weeks followed by 24 weeks of subsequent PEG-IFNα therapy, resulted in HBsAg clearance and HBV DNA negativity rates of 22% and 17%, respectively [20]. These rates declined to 9% and 15%, respectively, after 24 weeks following treatment cessation. Sequential treatment with bepirovirsen and PEG-IFNαwas found to significantly diminish relapse rates, with earlier commencement of sequential therapy associated with lower relapse rates.

These outcomes suggest that combination therapy, particularly involving bepirovirsen, could enhance therapeutic results for patients with CHB. This approach appears to be notably effective for NA-experienced patients or those with a heightened risk of relapse, as sequential therapy substantially lowers relapse rates, especially when initiated earlier. Bepirovirsen is currently progressing through global phase III clinical trials (B-Well 1) to further assess its effectiveness and safety, positioning it as a promising new therapeutic option for CHB.

#### HBsAg secretion inhibitors (Nucleic Acid Polymers – NAPs)

HBsAg secretion inhibitors reduce serum HBsAg by blocking its intracellular assembly and release, primarily in the ER-Golgi intermediate compartment (ERGIC). They disrupt the formation of 22-nm subviral particles (SVPs), largely derived from integrated HBV DNA, possibly by targeting host chaperones such as TMED2 [21].

Among NAPs, REP 2139 and REP 2165 are the most extensively studied. In a phase II trial (REP 401), 40 CHB patients received Tenofovir Disoproxil Fumarate (TDF) 300 mg QD for 24 weeks, then were randomized to TDF + PEG-IFNα (180 µg QW) with or without NAPs (REP 2139-Mg or REP 2165-Mg, 250 mg IV QW) for an additional 48 weeks. All patients in the control group later crossed over to receive the same triple therapy, allowing all 40 participants to complete 48 weeks of NAP-based treatment. At EOT, 60% of participants (24/40) achieved HBsAg levels ≤ 0.05 IU/mL, while 14 participants showed sustained HBsAg seroconversion and 13 maintained virologic control at 24 weeks post-treatment. Compared to the control group, NAP recipients showed significantly greater reductions in HBsAg and higher rates of seroconversion. ALT flares were more frequent in the NAP groups but were self-limited and asymptomatic [22].

The REP 401 study showed that adding NAPs to TDF + PEG-IFNα improves HBsAg clearance, particularly in patients with compensated liver function and no cirrhosis, though limited by small sample size and frequent ALT flares.

#### Gene editing technology

Gene-editing technologies represent a promising strategy for CHB by directly targeting HBV cccDNA, with the main findings summarized in Table 1 and additional details provided in the Appendix S3.

### Immune-targeting therapies

#### Toll-like receptor (TLR) agonists

TLR agonists activate innate immune cells, such as plasmacytoid dendritic cells and monocytes, inducing type I interferons (e.g., IFN-α) and proinflammatory cytokines (TNF-α, IL-6, IL-12), thereby enhancing antiviral responses and HBV-specific immunity [23].

Ruzotolimod, an oral TLR7 agonist, showed promise in preclinical studies by suppressing viral replication and inducing seroconversion, particularly when combined with ETV [24]. However, Phase II trials showed only 18% of patients achieved HBsAg seroclearance at EOT, and 12% maintained it at 24 weeks post-treatment [25]. GS-9688 activates monocytes and macrophages to enhance NK cells and HBV-specific T cells [26]. It also targets liver Kupffer cells, reshaping the immune microenvironment [27]. In a Phase II clinical trial, neither GS-9688 monotherapy nor its combination with TAF met the primary efficacy endpoint— a ≥ 1 log_10_ IU/mL reduction in HBsAg after 24 weeks of treatment [28]. TLR agonist combinations, though not superior in trials, remain a potential oral option for patients with poor adherence, NA resistance, or weak immunity.

#### Therapeutic vaccines

Therapeutic vaccines aim to overcome immune tolerance and restore virus-specific responses against HBsAg. The overall strategies and outcomes are summarized in Table 1, while detailed descriptions of vaccine types, clinical evaluations, and combination approaches are provided in the Appendix S3.

#### Immune checkpoint inhibitors(ICIs)

ICIs are monoclonal antibodies that block inhibitory receptors on T cells—primarily PD-1 or its ligand PD-L1—to reverse immune exhaustion. By disrupting these checkpoint pathways, ICIs restore HBV-specific T cell activity, enhance cytokine production, and reinvigorate cytotoxic responses, thereby promoting immune-mediated clearance of infected hepatocytes [29].

Envafolimab (ASC22), a humanized PD-L1 antibody, demonstrated encouraging outcomes in a Phase IIb trial with virologically suppressed and HBeAg-negative patients, showing an average HBsAg reduction of 0.309 log at 24 weeks [30]. Importantly, 43% (3 out of 7) of participants with an initial HBsAg level below 100 IU/mL achieved HBsAg clearance. Mild ALT elevations observed among these patients underscored the efficacy of the treatment.

In a separate Phase Ib study, 22 participants received 12 weeks of treatment with nivolumab (a PD-1 inhibitor), either alone or in combination with the therapeutic T-cell vaccine GS-4774. The results from the high-dose group indicated that 3 patients (13.6%) saw a reduction in HBsAg of more than 0.5 log at week 24, with one patient (4.5%) maintaining undetectable HBsAg levels for 12 months post-treatment [31]. PD-1 inhibitors alleviate T-cell suppression, and their integration with vaccines or antibody treatments further stimulates the host’s immune system, leading to enhanced HBsAg clearance.

This combined treatment strategy is notably effective for patients with low initial HBsAg levels and holds the potential to expedite HBsAg clearance, thereby contributing to the achievement of a functional cure.

#### Checkpoint-modified therapeutic vaccines

Checkpoint-modified therapeutic vaccines deliver HBV antigens alongside localized checkpoint blockade (e.g., anti-PD-1, anti-CTLA-4), reactivating exhausted HBV-specific T cells [32, 33].

In a Phase 1b study, VRON 0200 monotherapy was well tolerated in 27 NA-treated CHB patients, with no treatment-related SAEs over 8,000 patient-days. By Day 154, 27% showed HBsAg declines (–2.3 to –0.4 log₁₀ IU/mL) [34]. In a third cohort, VRON 0200 priming followed by elebsiran(siRNA) and tobevibart (anti-HBsAg monoclonal antibody) led to rapid HBsAg reductions (–3.6 to –1.3 log₁₀ IU/mL) in all evaluable patients (n = 6), with two achieving HBsAg loss and all seroconverting to HBsAb by Day 35 [35].

Compared to systemic checkpoint inhibitors, this approach enables targeted immune modulation with reduced toxicity and promotes memory T cell formation. VRON 0200 may serve as an effective immune primer in combination regimens, particularly for virally suppressed, non-cirrhotic CHB adults with baseline HBsAg < 500–1000 IU/mL seeking finite-duration functional cure [36].

#### Monoclonal antibodies

VIR-3434 is a neutralizing antibody that blocks HBV entry through surface antigen binding and reduces circulating HBsAg, thereby promoting immune reactivation [37]. Early-phase studies demonstrated antiviral efficacy, and in combination with VIR-2218 it achieved notable HBsAg loss [38,39]. Representative results are shown in Table 1, with full details provided in the Appendix S3.

#### Apoptosis inducer

Apoptosis inducers like APG-1387 restore TNF-α–mediated apoptosis in HBV-infected hepatocytes by neutralizing IAPs, reactivating the TNFR1–caspase pathway [40]. In a Phase I trial, 12 mg and 30 mg doses significantly reduced HBV DNA, HBsAg, and HBeAg, with sequential treatment showing better reductions than monotherapy [41]. A Phase II study with APG-1387 and ETV is ongoing. These findings suggest APG-1387 enhances T-cell responses and may improve antiviral therapy, especially when combined with NAs for a functional cure.

#### Gene-engineered T cells

Gene-engineered T cells are an emerging approach to enhance HBV-specific T cell responses. These T cells are modified to express chimeric antigen receptors (CARs) targeting anti-HBs or to incorporate HBV-specific T cell receptors (TCRs), enabling them to recognize and eliminate HBV-infected cells for a more targeted immune response [42].

SCG101 is an autologous TCR-T cell therapy targeting HBsAg-expressing hepatocytes via MHC presentation. In a Phase 1 trial of 18 advanced HBV-related HCC patients—all HLA-A*02:01( +), HBsAg ( +), and HBV DNA ≤ 1000 IU/mL—94% had prior NA therapy and 72% had baseline cirrhosis. A single infusion (5 × 10⁷ or 1 × 10⁸ cells/kg) resulted in a 1.0–4.6 log_10_ HBsAg reduction in 94% of patients within 28 days. Most maintained HBsAg < 100 IU/mL for up to one year, and 23.5% achieved complete clearance. These findings suggest SCG101 may serve as a key component in combination strategies for functional cure in difficult-to-treat cases [43, 44].

IMC-I109V, LT-V11, and ALVR107 are promising T cell therapies under early clinical evaluation, particularly in combination with antivirals such as bulevirtide. Relevant data and combination strategies are summarized in Table 1.

A comprehensive overview of all representative agents discussed in this review, including drug class, combination mechanisms, patient profiles, treatment outcomes, and safety data, is provided in Table S3 in Appendix S2.

## Challenges and future perspectives of combination therapy

Despite its potential, the implementation of combination therapy for CHB with novel drugs presents several challenges. These include uncertainties regarding efficacy, safety monitoring, drug resistance, and cost-effectiveness. A more detailed discussion of these issues is provided in Appendix (Appendix S4.)

## Limitations

This review lacks quantitative synthesis, limiting cross-trial comparisons. Most novel agents are still in early phases with limited long-term data. Although HBsAg loss is a surrogate for functional cure, its validity is debated. Broader frameworks integrating virological, biochemical, and immunological endpoints may better capture therapeutic impact in real-world CHB populations.

## Conclusions

With ongoing investment in drug development, many novel agents have already demonstrated significantly improved efficacy compared to current standard therapies. When used in combination, these drugs offer even greater efficacy in achieving a functional cure for CHB. However, optimizing drug efficacy remains challenging, particularly in patients with relatively high baseline HBsAg levels. Additional challenges include monitoring side effects from multiple drugs, managing potential drug resistance, and reducing the economic burden. By overcoming these barriers, combination therapy strategies hold the promise of substantially reducing the global burden of hepatitis B.

## Supplementary Information

Below is the link to the electronic supplementary material.

Supplementary material 1 (docx 82 KB)

## Data Availability

Not applicable.

## References

[1] World Health Organization. Global hepatitis report 2024: action for access in low-and middle-income countries. Geneva: World Health Organization; 2024.

[2] Devarbhavi H, Asrani SK, Arab JP, Nartey YA, Pose E, Kamath PS. Global burden of liver disease: 2023 update. J Hepatol. 2023;79:516–537 36990226 10.1016/j.jhep.2023.03.017

[3] Dusheiko G, Agarwal K, Maini MK. New approaches to chronic hepatitis B. N Engl J Med. 2023;388:55–69 36599063 10.1056/NEJMra2211764

[4] Lim SG, Baumert TF, Boni C, Gane E, Levrero M, Lok AS, et al. The scientific basis of combination therapy for chronic hepatitis B functional cure. Nat Rev Gastroenterol Hepatol. 2023;20:238–253 36631717 10.1038/s41575-022-00724-5

[5] Lucifora J, Protzer U. Attacking hepatitis B virus cccDNA—the holy grail to hepatitis B cure. J Hepatol. 2016;64:S41–S48 27084036 10.1016/j.jhep.2016.02.009

[6] Terrault NA, Lok AS, McMahon BJ, Chang KM, Hwang JP, Jonas MM, et al. Update on prevention, diagnosis, and treatment of chronic hepatitis B: AASLD 2018 hepatitis B guidance. Hepatology. 2018;67:1560–1599 29405329 10.1002/hep.29800 PMC5975958

[7] Wedemeyer H, Aleman S, Brunetto M, Blank A, Andreone P, Bogomolov P, et al. Bulevirtide monotherapy in patients with chronic HDV: efficacy and safety results through week 96 from a phase III randomized trial. J Hepatol. 2024;81(4):621–629 38734383 10.1016/j.jhep.2024.05.001

[8] Wedemeyer H, Aleman S, Brunetto MR, Blank A, Andreone P, Bogomolov P, et al. A phase 3, randomized trial of bulevirtide in chronic hepatitis D. N Engl J Med. 2023;389(1):22–32 37345876 10.1056/NEJMoa2213429

[9] Wedemeyer H, Schöneweis K, Bogomolov P, Blank A, Voronkova N, Stepanova T, et al. 2023 Safety and efficacy of bulevirtide in combination with tenofovir disoproxil fumarate in patients with hepatitis B virus and hepatitis D virus coinfection (MYR202): a multicentre, randomised, parallel-group, open-label, phase 2 trial. Lancet Infect Dis. 2023;23(1):117–129 36113537 10.1016/S1473-3099(22)00318-8

[10] Zoulim F, Zlotnick A, Buchholz S, Donaldson E, Fry J, Gaggar A, et al. Nomenclature of HBV core protein-targeting antivirals. Nat Rev Gastroenterol Hepatol. 2022;19(12):748–750 36207612 10.1038/s41575-022-00700-z PMC10442071

[11] Zhao N, Jia B, Zhao H, Xu J, Sheng X, et al. A first-in-human trial of GLS4, a novel inhibitor of hepatitis b virus capsid assembly, following single- and multiple-ascending-oral-dose studies with or without ritonavir in healthy adult volunteers. Antimicrob Agents Chemother. 2019. 10.1128/AAC.01686-19 31636065 10.1128/AAC.01686-19 PMC7187578

[12] Zhang H, Wang F, Zhu X, Chen Y, Chen H, Li X, et al. Antiviral activity and pharmacokinetics of the hepatitis B virus (HBV) capsid assembly modulator GLS4 in patients with chronic HBV infection. Clin Infect Dis. 2021;73(2):175–182 32649736 10.1093/cid/ciaa961 PMC8516514

[13] Yuen MF, Asselah T, Jacobson IM, Brunetto MR, Janssen HL, Takehara T, et al. Efficacy and safety of the siRNA JNJ-73763989 and the capsid assembly modulator JNJ-56136379 (bersacapavir) with nucleos(t)ide analogues for the treatment of chronic hepatitis B virus infection (REEF-1): a multicentre, double-blind, active-controlled, randomised, phase 2b trial. Lancet Gastroenterol Hepatol. 2023;8(9):790–802 37442152 10.1016/S2468-1253(23)00148-6

[14] Nguyen L, Nguyen TT, Kim JY, Jeong JH. Advanced siRNA delivery in combating hepatitis B virus: mechanistic insights and recent updates. J Nanobiotechnology. 2024;22(1):745 39616384 10.1186/s12951-024-03004-3 PMC11608496

[15] Yuen MF, Schiefke I, Yoon JH, Ahn SH, Heo J, Kim JH, et al. RNA interference therapy with ARC-520 results in prolonged hepatitis B surface antigen response in patients with chronic hepatitis B infection. Hepatology. 2020;72(1):19–31 31654573 10.1002/hep.31008 PMC7496196

[16] Hou J, Zhang W, Xie Q, Hua R, Tang H, Morano Amado LE, et al. Xalnesiran with or without an immunomodulator in chronic hepatitis B. N Engl J Med. 2024;391(22):2098–2109 39774313 10.1056/NEJMoa2405485

[17] Yuen MF, Lim YS, Yoon KT, Lim TH, Heo J, Tangkijvanich P, et al. VIR-2218 (elebsiran) plus pegylated interferon-alfa-2a in participants with chronic hepatitis B virus infection: a phase 2 study. Lancet Gastroenterol Hepatol. 2024;9(12):1121–1132 39389081 10.1016/S2468-1253(24)00237-1

[18] Yuen MF, Heo J, Jang JW, Yoon JH, Kweon YO, Park SJ, et al. Safety, tolerability and antiviral activity of the antisense oligonucleotide bepirovirsen in patients with chronic hepatitis B: a phase 2 randomized controlled trial. Nat Med. 2021;27(10):1725–1734 34642494 10.1038/s41591-021-01513-4 PMC8516644

[19] Yuen MF, Lim SG, Plesniak R, Tsuji K, Janssen HL, Pojoga C, et al. Efficacy and safety of bepirovirsen in chronic hepatitis B infection. N Engl J Med. 2022;387(21):1957–1968 36346079 10.1056/NEJMoa2210027

[20] Buti M, Heo J, Tanaka Y, Andreone P, Atsukawa M, Cabezas J, et al. Sequential Peg-IFN after bepirovirsen may reduce post-treatment relapse in chronic hepatitis B. J Hepatol. 2025;82(2):222–234 39214467 10.1016/j.jhep.2024.08.010

[21] Boulon R, Blanchet M, Lemasson M, Vaillant A, Labonté P. Characterization of the antiviral effects of REP 2139 on the HBV lifecycle *in vitro*. Antiviral Res. 2020;183:104853 32585322 10.1016/j.antiviral.2020.104853

[22] Bazinet M, Pântea V, Placinta G, Moscalu I, Cebotarescu V, Cojuhari L, et al. Safety and efficacy of 48 weeks REP 2139 or REP 2165, tenofovir disoproxil, and pegylated interferon Alfa-2a in patients with chronic HBV infection Naïve to Nucleos(t)ide therapy. Gastroenterology. 2020;158(8):2180–2194 32147484 10.1053/j.gastro.2020.02.058

[23] Du Y, Wu J, Liu J, Zheng X, Yang D, Lu M. Toll-like receptor-mediated innate immunity orchestrates adaptive immune responses in HBV infection. Front Immunol. 2022;13:965018 35967443 10.3389/fimmu.2022.965018 PMC9372436

[24] Wildum S, Korolowicz KE, Suresh M, Steiner G, Dai L, Li B, et al. Toll-like receptor 7 agonist RG7854 mediates therapeutic efficacy and seroconversion in woodchucks with chronic hepatitis B. Front Immunol. 2022;13:884113 35677037 10.3389/fimmu.2022.884113 PMC9169629

[25] Lok ASF. Toward a functional cure for hepatitis B. Gut Liver. 2024;18(4):593–601 38533651 10.5009/gnl240023 PMC11249939

[26] Amin OE, Colbeck EJ, Daffis S, Khan S, Ramakrishnan D, Pattabiraman D, et al. Therapeutic potential of TLR8 agonist GS-9688 (Selgantolimod) in chronic hepatitis B: remodeling of antiviral and regulatory mediators. Hepatology. 2021;74(1):55–71 33368377 10.1002/hep.31695 PMC8436741

[27] Roca Suarez AA, Plissonnier ML, Grand X, Michelet M, Giraud G, Saez-Palma M, et al. TLR8 agonist selgantolimod regulates Kupffer cell differentiation status and impairs HBV entry into hepatocytes via an IL-6-dependent mechanism. Gut. 2024;73(12):2012–2022 38697771 10.1136/gutjnl-2023-331396 PMC12210347

[28] Daffis S, Balsitis S, Chamberlain J, Zheng J, Santos R, Rowe W, et al. Toll-like receptor 8 agonist GS-9688 induces sustained efficacy in the woodchuck model of chronic hepatitis B. Hepatology. 2021;73(1):53–67 32246499 10.1002/hep.31255 PMC7898792

[29] Balsitis S, Gali V, Mason PJ, Chaniewski S, Levine SM, Wichroski MJ, et al. Safety and efficacy of anti-PD-L1 therapy in the woodchuck model of HBV infection. PLoS One. 2018;13(2):e0190058 29444087 10.1371/journal.pone.0190058 PMC5812555

[30] Qian J, Xie Y, Mao Q, Xie Q, Gu Y, Chen X, et al. A randomized phase 2b study of subcutaneous PD-L1 antibody ASC22 in virally suppressed patients with chronic hepatitis B who are HBeAg-negative. Hepatology. 2025;81(4):1328–1342 38976867 10.1097/HEP.0000000000001006

[31] Gane E, Verdon DJ, Brooks AE, Gaggar A, Nguyen AH, Subramanian GM, et al. Anti-PD-1 blockade with nivolumab with and without therapeutic vaccination for virally suppressed chronic hepatitis B: a pilot study. J Hepatol. 2019;71(5):900–907 31306680 10.1016/j.jhep.2019.06.028

[32] Hoogeveen RC, Boonstra A. Checkpoint inhibitors and therapeutic vaccines for the treatment of chronic HBV infection. Front Immunol. 2020;11: 401 32194573 10.3389/fimmu.2020.00401 PMC7064714

[33] Gane EJ, Currie S, Wong G. HBsAg declines and immune responses following a single dose of VRON-0200: interim results from a Phase 1b study for HBV functional cure in chronic HBV-infected patients. Paper presented at: EASL Congress 2024; 2024/06/05, 2024; Amsterdam, Netherlands.

[34] Gane EJ, Wong GLH, Currie S. HBsAg declines, and T cell increases, observed in CHB patients: Interim results from P1b trial of VRON‑0200 following prime-only and prime‑boost dosing (Abstract #1404). Paper presented at: EASL Congress 2025; 2025/05/07, 2025; Amsterdam, Netherlands.

[35] Gane EJ, Wong GLH, Currie S. Rapid HBsAg declines and HBsAb seroconversion observed with a single dose of VRON‑0200 plus Tobevibart and Elebsiran: preliminary results of a VRON‑0200 combination treatment (Late‑Breaker Abstract LBP‑033). Paper presented at: EASL Congress 2025; 2025/05/07, 2025; Amsterdam, Netherlands.

[36] Virion T. Virion therapeutics reports new data from two clinical presentations at EASL 2025, including rapid and profound hepatitis B surface antigen declines. *EIN Presswire.* 2025.

[37] Beretta M, Mouquet H. Advances in human monoclonal antibody therapy for HBV infection. Curr Opin Virol. 2022;53: 101205 35123237 10.1016/j.coviro.2022.101205

[38] Lempp FA, Volz T, Cameroni E, Benigni F, Zhou J, Rosen LE, et al. Potent broadly neutralizing antibody VIR-3434 controls hepatitis B and D virus infection and reduces HBsAg in humanized mice. J Hepatol. 2023;79:1129–1138 37459920 10.1016/j.jhep.2023.07.003

[39] Agarwal K. A phase 1 study evaluating the neutralizing, vaccinal monoclonal antibody VIR-3434 in participants with chronic hepatitis B virus infection. Proceedings of the Hepatitis B: Novel Therapeutic Approaches (Viral Hepatitis); 2021/06/25; New York, NY, USA.

[40] Chen Z, Chen J, Liu H, Dong W, Huang X, Yang D, et al. The SMAC mimetic APG-1387 sensitizes immune-mediated cell apoptosis in hepatocellular carcinoma. Front Pharmacol. 2018;9:1298 30459627 10.3389/fphar.2018.01298 PMC6232623

[41] Ascentage P. First-in-Human Study of APG-1387, Targeting Inhibitor of Apoptosis Proteins, For the Treatment of Patients with Chronic Hepatitis B. 73rd American Association for the Study of Liver Diseases Annual Meeting (AASLD 2022); 2022/11/07.

[42] Bertoletti A, Tan AT. Engineering HBV-specific T cells for the treatment of HBV-related HCC and HBV infection: past, present, and future. Clin Mol Hepatol. 2024;30(4):728–734 38934109 10.3350/cmh.2024.0469 PMC11540402

[43] Du S, Sun H, Hou J, Huang X, Cui J, Wang N, et al. SCG101 HBV-specific TCR-T cell therapy demonstrates dual antiviral and antitumor activities, achieving HBV clearance in liver biopsies and functional cure in HBV-related hepatocellular carcinoma patients. J Hepatol*.* 2025;82(Supplement 1):Abstract LBP-017.

[44] Wan X, Wisskirchen K, Jin T, Yang L, Wang X, Wu XA, et al. Genetically-modified, redirected T cells target hepatitis B surface antigen-positive hepatocytes and hepatocellular carcinoma lesions in a clinical setting. Clin Mol Hepatol. 2024;30(4):735–755 38808361 10.3350/cmh.2024.0058 PMC11540345

