# Supplementary material for: Combination therapies for chronic hepatitis B in the era of emerging novel drugs
Source: Hepatol Int. 2025 Oct 17;20(1):9–18. doi: 10.1007/s12072-025-10888-2 (PMC12923412; doi:10.1007/s12072-025-10888-2)
Supplement: Supplementary file 1 — Supplementary material 1 (docx 82 KB) [file 12072_2025_10888_MOESM1_ESM.docx]

Supplementary Appendix

**Appendix S1: Chronic HBV infection, standard treatment and functional cure**

HBV infects hepatocytes by leveraging the cell-surface sodium taurocholate cotransporting polypeptide (NTCP) as its entry receptor. Once inside the cell, the viral genome forms covalently closed circular DNA (cccDNA) in the nucleus, which serves as a template for producing viral RNAs—including pre-genomic RNA (pgRNA) and subgenomic RNAs—and proteins such as HBsAg [1]. The formation of cccDNA establishes a stable reservoir that allows the virus to evade immune clearance and sustain chronic infection. Additionally, HBV DNA can integrate into the host genome, providing an alternative and independent source of HBsAg production [2–4]. Nucleoside and nucleotide analogues (NAs) are the cornerstone of anti-HBV treatment by competitively inhibiting the HBV polymerase, thereby disrupting the synthesis of HBV DNA, achieving near-complete viral suppression in most adherent patients. Long-term use of these therapies has demonstrated benefits, including reduced liver fibrosis progression and a decrease in liver-related complications [5]. However, these agents have little impact on cccDNA activity or HBsAg production. As a result, the reservoir persists and underpins the continued production of viral antigens and contributes to the residual risk of HCC, even in patients with effective viral suppression [6, 7].

Pegylated interferon alfa (Peg-IFNα) has also been approved for the anti-HBV treatment [8]. The main mechanism of action of Peg-IFNα is its antiviral effects by modulating both innate and adaptive immune responses and can facilitate the eradication of cccDNA by acting at various stages of the viral life cycle [9]. Comparing to NAs alone, the combination of NAs and Peg-IFNα has demonstrated a significantly higher rate of undetectable HBsAg after HBV DNA eradication [10]. Off-treatment sustained undetectable HBV DNA and HBsAg has been referred as “functional cure”, which virtually eliminate the long-term risks associated with chronic HBV infection and becomes the therapeutic focus at the current stage [11, 12].

However, the clinical application of Peg-IFNα is restricted due to a range of side effects, and the combinative strategy with NAs and PegIFNα is unsatisfactory in achieving a functional cure, which remains a critical unmet goal in HBV management [13–15].

**Appendix S2: Supplementary Tables**

| **Table S1. Direct-acting antiviral agents targeting different stages of the HBV lifecycle** | | | |
| --- | --- | --- | --- |
| **Type and compound** | **Originator** | **Phases** | **Reference number** |
| **Entry inhibitors** | | |  |
| Hepalatide | Shanghai HEP Pharmaceutical Co.Ltd. China | II | NCT04426968 |
| Hepcludex (bulevirtide) | Gilead Sciences, USA | III | NCT03852719 |
| **Capsid assembly modulators** | | |  |
| Morphothiadin (GLS4) | HEC Pharma, China | IIIa | NCT04147208 |
| QL-007 | Qilu, China | II | NCT04157699 |
| EDP-514 | Enanta Pharma, USA | II | NCT04470388,NCT04008004 |
| GST-HG141 | Cosunter Pharmaceutical Co., Ltd., Fujian, China | II | NCT04470388 |
| ZM-H1505R | ZhiMeng Biopharma, China | IIa | NCT05637541 |
| ABIH3733 | Assembly Biosciences, USA | Ib | NCT05414981 |
| ALG-000184 | Aligos Therapeutics, USA | II | NCT04536337NCT06963710 |
| **Small interfering RNAs** | | |  |
| Xalnesiran(RG6346) | Roche with Dicerna, USA | IIb | NCT04225715 |
| VIR2218 | Alnylam and Vir Biotechnology, USA, with Brii Biosciences, China | II | NCT04412863,NCT04856085 |
| JNJ3989 (ARO-HBV) | Arrowhead Pharma, USA, with Janssen | IIb | NCT03982186 |
| ALG-125755 | Aligos Therapeutics, USA | I | NCT05561530 |
| AB-729 | Arbutus Biopharma, USA | II | NCT06277037,NCT04980482 |
| STSG-0002 | Staidson, China | II | NCT04507269,NCT05760703 |
| RBD1016 | Ribo Life Science, China | II | NCT05961098 |
| **Antisense oligonucleotides** | | |  |
| Bepirovirsen (GSK3228836) | Ionis Pharma, USA with GSK | III | NCT05630820,NCT05630807 |
| AHB-137 | AusperBio, China | IIb | NCT06550128 |
| **HBsAg secretion inhibitors** | | |  |
| REP 2139 | Replicor, Canada | II | NCT02565719 |
| REP 2165 | Replicor, Canada | II | NCT02233075 |
| GST-HG131 | Cosunter Pharmaceutical, China | IIa | NCT06263959 |
| LP-128 | Lupeng Pharmaceutical, China | I | NCT05130567 |
| GST-HG121 | Cosunter Pharmaceutical, China | I | NCT05576584 |
| **Gene editing technology** | |  |  |
| PBGENE-HBV(ARCUS) | Precision BioSciences, USA | I | NCT06680232 |
| EBT107(CRISPR/Cas 9) | Editas Medicine, USA | Preclinical phase | / |
| *HBsAg: Hepatitis B surface antigen; HBV: Hepatitis B virus; | |  |  |
|  |  |  |  |
| **Table S2. Novel antiviral immunomodulators currently under clinical evaluation** | | | |
| **Type and compound** | **Originator** | **Phase** | **Reference number** |
| **Toll like receptor** | | |  |
| Selgantolimod (GS9688) | Gilead Sciences, USA | II | NCT03615066,NCT03491553 |
| Ruzotolimod(RG7854) | Roche, Switzerland | II | NCT04225715 |
| TQA3334 | ChiaTai TianQing, China | II | NCT04202653,NCT04180150 |
| HRS9950 | Hengrui Pharmaceuticals, China | II | NCT05905458 |
| **Therapeutic vaccines** | | |  |
| CVI-HBV-002 | CHA Vaccine Research Institute Co., Ltd., Korea | IIb | NCT04289987 |
| GS-4774 | Gobelmmune with Gilead, USA | II | NCT01943799,NCT02174276 |
| HepTcell | Altimmune, USA | II | NCT04684914 |
| VVX001 | Viravaxx, Austria | II | NCT03625934 |
| VBI-2601 (BRII-179) | VBI Vaccines, USA, with Brii Biosciences | II/IIb | NCT04749368,NCT05970289 |
| GSK3528869A | GSK Biologicals, UK | II | NCT05276297 |
| VTP-300 | Vaccitech, USA | IIb | NCT05343481 |
| ISA104 | ISA Pharma, The Netherlands | I/II | NCT05841095 |
| **Immune checkpoint inhibitors** | | |  |
| ASC22 (KN035) | Ascletis Pharma, PR China | IIb | NCT04568265 |
| RG6084 (RO7191863) | Roche, Switzerland | II | NCT04225715 |
| AB-101 | Arbutus Biopharma, USA | I | NCT05960240 |
| **Checkpoint-modified therapeutic vaccines** | |  |  |
| VRON 0200 | Virion Therapeutics, LLC | Ib | NCT06070051 |
| AdC6‑gDHBV2 | Virion Therapeutics / Wistar Inst. | Preclinical phase | / |
| **Monoclonal antibodies** | | |  |
| GC1102 | Green Cross, South Korea | II | NCT03801798; |
| VIR-3434 (BRII-877) | Vir Biotech, USA, with Brii Biosciences, China | II | NCT04856085 |
| **Apoptosis inducer** |  |  |  |
| APG‑1387 | Ascentage Pharma, China | II | NCT04568265 |
| CRV 431 | ConteaVir ,USA | I | NCT03596697 |
| **Gene-engineered T cells** | |  |  |
| SCG101‑V | SCG Cell Therapy, China | I /II | NCT05417932 |
| IMC-I109V | Immunocore, UK/USA | I | NCT05867056 |
| LT-V11 | Immunocore, UK/USA | Preclinical phase | / |
| ALVR107 | AlloVir, Inc.，USA | Preclinical phase | / |

**Table S3 Combination approaches targeting CHB functional cure**

|  | | | | | | |  |  |
| --- | --- | --- | --- | --- | --- | --- | --- | --- |
| **Drug Class** | **Representative Agent and Combined Protocols** | **Combination mechanism** | **Target Population / Patient Characteristics** | **Treatment Duration** | **EOT Key Events (HBV DNA ↓, HBsAg ↓/Clearance)** | **Functional Cure (HBsAg Loss, 24w Off-Tx)** | **Main adverse events** |  |
| Entry Inhibitor | **Bulevirtide**±Peg-IFNα | Entry blockade + immune stimulation (via PEG-IFNα) | HDV co-infection; HBsAg low-level CHB; immunoactive stage | 96 weeks + 48weeks follow-up | HBsAg ↓ ≥1 log in 16% (2 mg) and 12% (10 mg) at EOT | HBsAg loss: 8%(2 mg); 4%(10 mg) ; none with monotherapy | Injection site reactions, flu-like symptoms, ALT flares |  |
| CAM-A | **GLS4** + Ritonavir + Entecavir | Capsid destabilization + NA-mediated viral suppression | HBeAg-positive CHB patients; includes treatment-naïve individuals and those previously on long-term entecavir with virological suppression | 96 weeks + 48week follow-up | Treatment-naïve: HBV DNA and HBsAg LSM reduction at W48: −6.28/−0.87 vs −5.72/−0.65 log₁₀ IU/mL (combo vs mono);  Virally suppressed: pgRNA and HBsAg LSM reduction: −1.61/−0.17 vs −0.28/−0.06 log₁₀ IU/mL; none HBsAg loss. | not reported | ALT elevation, hypertriglyceridemia |  |
| CAM-E | **JNJ-6379** + NA ± siRNA(JNJ-3989) | Capsid assembly inhibition + RNAi-enhanced antigen reduction | Early/immune-tolerant CHB; triple therapy for HBeAg−/suppressed | 48Weeks+48Weeks follow-up | JNJ-3989 + NA (dual): HBV DNA ↓ (marked, dose-dependent); HBsAg ↓ –1.5 to –2.6 log; HBsAg loss 1–3% / 0% seroconv.  JNJ-6379 + NA (dual): HBV DNA ↓ (mild); HBsAg ↓ –0.07 log; HBsAg loss 0%  JNJ-3989 + JNJ-6379 + NA (triple): HBV DNA ↓ (dose-dependent, sustained post-treatment); HBsAg ↓ –1.8 log; HBsAg loss 0% at EOT | JNJ-3989 + NA (dual): HBsAg ↓ –1.0 to –1.9 log (24W off-Tx); 3 pts cleared HBsAg; 1 pt met full functional cure (off NA); others remained on NA, HBeAg/HBcrAg+  JNJ-6379 + NA (dual): HBsAg ↓ –0.15 log (24W off-Tx); No HBsAg loss; No functional cure  JNJ-3989 + JNJ-6379 + NA (triple): HBsAg ↓ –1.4 log (24W off-Tx); No HBsAg loss; No functional cure | headache, nausea, decrease in eGFR |  |
| CAM (Class II / Hybrid) | ALG-000184 (monotherapy or + entecavir) | Dual capsid targeting + NA-mediated suppression | Capsid assembly inhibition; hybrid Class I/II activity | 12–48 weeks | HBV DNA ↓ up to 4.2 log₁₀; HBsAg ↓ up to 0.8 log₁₀ with monotherapy; up to 1.65 log₁₀ with combo | Not reported; | ALT elevation (transient, immune-related) |  |
| siRNA | Xalnesiran + Peg-IFNα-2a + NA | Antigen silencing + immune activation (PEG-IFNα) | Virally suppressed, patients with low HBsAg (<1000 IU/mL),HBeAg-negative status aimed at achieving functional cure. | 48W+48Wfollow-up | HBsAg loss / seroconversion at EOT: 3–7% / 3% (mono); 30% / 23% (como) ;HBV DNA <10 IU/mL in most patients | HBsAg loss / seroconversion : 3–7% / 0~3% (mono); 23% / 23% (como) ;HBV DNA <10 IU/mL in most patients | ALT elevation  Flu-like symptoms  Injection-site reaction |  |
| siRNA | **VIR-2218** + Peg-IFNα | Extended antigen reduction + immune reactivation | NRTI-treated, non-cirrhotic patients with moderate HBsAg burden | 12-48 weeks | HBsAg ↓ up to –3.0 log₁₀; loss in 28–31%,no HBV DNA rebound | HBsAg loss 持续28–31%,; anti-HBs in 91% of seroclearers | ALT elevation  Flu-like symptoms  Injection-site reaction |  |
| ASO | **Bepirovirsen** + NA ± PEG-IFNα | mRNA degradation + NA-based viral replication control | HBsAg ≤3000 IU/mL, HBeAg-negative, NA-experienced or naïve | 24–48 W | Bepirovirsen ± NA (B-Clear Study, 300 mg × 24w) EOT: HBsAg <LLOD + HBV DNA <LLOQ → 26% (On-NA), 29% (Not-on-NA)  Bepirovirsen → PEG-IFNα (B-Together Study, NA-experienced) EOT: HBsAg loss + HBV DNA undetectable →  • 22% (BPV 24w → PEG-IFNα 24w)  • 17% (BPV 12w → PEG-IFNα 24w) | Bepirovirsen ± NA (B-Clear Study, 300 mg × 24w) At 24w Off-Tx: HBsAg <LLOD + HBV DNA <LLOQ in ~9–10%  Bepirovirsen → PEG-IFNα (B-Together Study, NA-experienced) At 24w Off-Tx: HBsAg loss + HBV DNA undetectable:  • 9% (BPV 24w → PEG-IFNα 24w)  • 15% (BPV 12w → PEG-IFNα 24w) Earlier PEG-IFNα switch associated with lower relapse rates | Injection-site reaction，Fever，Fatigue，ALT elevation |  |
| HBsAg secretion inhibitors | **REP 2139/2165** + TDF + Peg-IFNα | Secretion blockade + viral suppression + immune stimulation | Suppressed HBV DNA but persistent HBsAg Compensated liver, no cirrhosis, good pegIFN tolerance High baseline HBsAg, normal/mild ALT, genotype D | 48 weeks (24w TDF lead-in + 48w triple therapy) | 60% achieved HBsAg ≤0.05 IU/mL; significant HBsAg reduction and seroconversion. | 14 (35%) achieved HBsAg seroconversion; 13 (32.5%) maintained virologic control at 24w off-Tx | Frequent ALT flares (some >1000 U/L), self-limited, asymptomatic |  |
| Gene Editing | CRISPR/Cas9 + RNAi/NA | cccDNA disruption + transcript knockdown | Preclinical stage; tested in hepatoma cells, primary hepatocytes, and animal models | Not yet defined; experimental dosing in models | HBV DNA↓, HBsAg↓(20–30%), cccDNA↓(up to 70%); HBsAg undetectable in mice (RNAi), DHBV DNA↓97–98% (Entecavir), cccDNA clearance Cas9-driven | Not yet demonstrated in humans | Unknown; human safety profile not established |  |
| Gene Editing | PBGENE-HBV | Targeted ARCUS-mediated gene disruption of HBV reservoir | early-phase trial population; non-cirrhotic | 2 doses (0.2 mg/kg), ongoing dose escalation | HBsAg ↓ ≥0.3 log₁₀ in 2/3 patients at 4 weeks; preclinical cccDNA ↓ 0.5 log₁₀ | Not yet achieved; functional cure under investigation | No dose-limiting toxicities reported in low-dose cohort |  |
| TLR-7 Agonists | Ruzotolimod + Xalnesiran | Innate immune stimulation + antigen reduction | early-phase study,HBsAg low-level | 48 weeks | 18% achieved HBsAg seroclearance at EOT | 12% maintained HBsAg loss at 24 weeks post-treatment | Mild to moderate flu-like symptoms; manageable |  |
| TLR-8 Agonists | GS-9688 ± TAF | Myeloid cell activation + antiviral cytokine induction | virally suppressed | 24 weeks | Did not achieve ≥1 log HBsAg ↓; no significant HBsAg clearance | Not achieved | GI symptoms, ALT elevations; generally mild |  |
| Protein-based vaccines | VBI-2601 + VIR-2218 | Immune priming + antigen clearance via siRNA | virally suppressed; early-phase | Multiple doses; duration varies by protocol | HBsAg ↓ 1.7–1.8 log IU/mL; Anti-HBs seroconversion in >30% | Not achieved; under evaluation | Mild injection site reactions; well tolerated |  |
| Epitope peptide-based vaccines | GS-4774 + Tenofovir | HBV-specific CD8+T cell stimulation + NA induced suppression | on NA therapy; virally suppressed | 20-24 weeks | No significant HBsAg ↓; strong CD8+ T-cell cytokine response (↑ IFN-γ, TNF, IL-2) | Not observed | Mild; no serious AEs reported |  |
| DNA-based vaccines | VTP-300 + Nivolumab / AB-729 | T cell priming + checkpoint-enhanced immune reactivation | often with baseline HBsAg <100 IU/mL | Sequential (e.g., AB-729 → VTP-300 ± nivolumab)≥48weeks | 29% had >1 log HBsAg ↓ with VTP-300 + nivolumab; 97% maintained HBsAg <100 IU/mL post AB-729 | Infrequent; under study | Mostly mild; immune-related risks with checkpoint inhibitors |  |
| Checkpoint-modified vaccines | VRON 0200 + Elebsiran + Tobevibart | Local checkpoint blockade + immune reprogramming | Virally suppressed CHB adults (non-cirrhotic), HBsAg <500–1000 IU/mL | VRON-0200 prime 28d + monthly Elebsiran & Tobevibart (6 doses) | Rapid HBsAg ↓ (–3.6 to –1.3 log₁₀ IU/mL by Day 35); all patients <10 IU/mL; universal HBsAb seroconversion | 2/6 achieved HBsAg loss (<0.05 IU/mL) by Day 140 | No treatment-related SAEs; Grade 1–2 TRAEs (e.g., rash); well tolerated overall |  |
| ICI（PD-L1） | Envafolimab | T cell reinvigoration via PD-L1 blockade | HBeAg-negative, virally suppressed CHB; baseline HBsAg <100 IU/mL (subgroup) | 24 weeks | Mean HBsAg ↓ 0.309 log; 43% (3/7) with baseline HBsAg <100 IU/mL achieved HBsAg clearance | not reported | Mild ALT elevations; well tolerated |  |
| ICI（PD-1） | Nivolumab ± GS-4774 | T cell reinvigoration + vaccine-primed response | virally suppressed | 12 weeks | 13.6% had >0.5 log HBsAg ↓ by Week 24; 1 patient (4.5%) maintained HBsAg loss at 12 months | 1/22 achieved sustained HBsAg loss | Generally mild; immune activation risk (e.g., ALT flares) |  |
| Monoclonal antibodies | VIR-3434 + VIR-2218 (± Peg-IFNα) | Antigen clearance + siRNA-based suppression ± immune modulation | treatment-naïve and -experienced; early-phase | 20–24 weeks | Single-dose VIR-3434: HBsAg ↓ ~1.8 log, HBV DNA ↓ ~2.0 log; Combo with VIR-2218: ~15% achieved HBsAg loss; | durability under evaluation | Mostly mild; no serious adverse events reported |  |
| Apoptosis inducer | APG-1387 ± Entecavir | Apoptotic sensitization of infected hepatocytes + viral suppression | HBeAg-positive; early-phase trial | 4–16 weeks; sequential NA combination | Monotherapy: HBV DNA ↓ 0.38 log₁₀ IU/mL (Day 28);  Sequential combo: HBV DNA ↓ 4.69 log₁₀, HBsAg ↓ 1.06 log₁₀, HBeAg ↓ 1.73 log₁₀ (Day 112) | Not yet achieved; functional cure under evaluation | Generally well tolerated; no severe AEs reported in Phase I |  |
| Gene-engineered T cells | SCG101±NAs | HBV-specific cytotoxicity via engineered TCR + NA support | Advanced HBV-related HCC; HLA-A*02:01(+), HBsAg(+), HBV DNA ≤1000 IU/mL; 94% on prior NA; 72% with cirrhosis | Single infusion of SCG101 + ongoing NA | 94% had 1.0–4.6 log₁₀ HBsAg reduction at 28 days; DNA already suppressed | 23.5% achieved complete HBsAg clearance (up to 1 year) | ALT elevation, mild CRS, cytopenia |  |
| **Abbreviations:** CAMs, Capsid Assembly Modulators; CAM-A, Class I CAM; CAM-E, Class II CAM; siRNA, Small Interfering RNA; ASO, Antisense Oligonucleotide; HBsAg, Hepatitis B Surface Antigen; HBV DNA, Hepatitis B Virus DNA; cccDNA, Covalently Closed Circular DNA; TLR, Toll-Like Receptor; ICI, Immune Checkpoint Inhibitor; PD-1, Programmed Cell Death Protein 1; PD-L1, Programmed Death-Ligand 1; EOT, End of Treatment; Tx, Treatment; w, week(s); ALT, Alanine Aminotransferase; NA(s), Nucleos(t)ide Analogues; IFN-α, Interferon-alpha; LLOQ, Lower Limit of Quantification. | | | | | | | |  |
|  |  |  |  |  |  |  |  |  |
|  |  |  |  |  |  |  |  |  |

**Appendix S3. Supplementary Results**

***Capsid assembly modulators (CAMs)***

**Clinical trial results of GLS4/RTV with or without entecavir (ETV)**

**Phase Ib Trial of GLS4/RTV**

A phase Ib study demonstrated that combining 240 mg of GLS4 with RTV led to a reduction in HBV DNA of 2.13 log₁₀ IU/mL over 28 days, compared to 3.5 log₁₀ IU/mL with entecavir (ETV) monotherapy, while reductions in HBsAg were 0.14 log₁₀ IU/mL and 0.33 log₁₀ IU/mL, respectively [16].

**Phase 2b Trial of GLS4/RTV + ETV**

In a phase 2b trial, GLS4/RTV combined with ETV demonstrated greater antiviral activity than ETV monotherapy in both treatment-naïve and virally suppressed patients with chronic hepatitis B.

- Treatment-naïve patients:
  At week 48, least-squares mean (LSM) reductions in HBV DNA and pgRNA were significantly greater in the GLS4/RTV + ETV group (−6.28 and −3.83 log₁₀, respectively) compared to the ETV group (−5.72 and −1.91 log₁₀, respectively). HBsAg also declined more with combination therapy (−0.87 vs −0.65 log₁₀ IU/mL).
- Virally suppressed patients:
  pgRNA and HBsAg levels declined more with combination therapy (−1.61 and −0.17 log₁₀) versus monotherapy (−0.28 and −0.06 log₁₀), although no HBsAg loss was achieved in either group at week 48 [17].

**Clinical trial results of Bersacapavir (JNJ-6379) in the REEF-1 study**

In the dual therapy group (JNJ-6379 + NA), the mean HBsAg decline was only −0.07 log₁₀ IU/mL at week 48, further decreasing to −0.15 log₁₀ IU/mL at week 72, indicating weak and unsustained antigen suppression. Moreover, no patients in this group met the predefined NA discontinuation criteria (ALT < 3 × upper limit of normal [ULN], HBV DNA below the lower limit of quantification [LLOQ], HBeAg negativity, and HBsAg < 10 IU/mL) at week 48, and no cases of functional cure were observed [18].

***Small interfering RNA (siRNA).***

siRNA-based therapies suppress HBV replication and protein production by degrading all viral transcripts from cccDNA or integrated DNA. siRNAs, processed by Dicer and loaded into the RISC complex, selectively target HBV mRNAs [19,20].

In a phase 2 trial of virally suppressed patients—of whom approximately 70% were HBeAg negative—on stable NA therapy, xalnesiran monotherapy led to limited HBsAg loss (3–7% at EOT). In contrast, combination regimens with ruzasvir or Peg-IFNα showed higher efficacy: HBsAg loss at EOT was 18% and 30%, and remained at 12% and 23% at W24, respectively. HBsAg seroconversion was observed in up to 23% by EOT, sustained in 20% and 17% at W24 and W48 in the Peg-IFNα-2a arm [21]. HBsAg clearance and seroconversion occurred only in patients with baseline HBsAg <1000 IU/mL, suggesting that such regimens are most effective in low-antigen, HBeAg-negative individuals.

Agents such as JNJ-3989, VIR-2218, RG-6346, and AB-729 have demonstrated 2–3 log₁₀ HBsAg reductions after 3–4 doses, with effects lasting 6–9 months [22-26]. However, monotherapy rarely achieves HBsAg clearance, and reductions tend to plateau. Greater reductions in HBsAg levels have been observed with combination therapies, such as VIR-2218 combined with PEG-IFNα or JNJ-3989 with NAs [27,28]. In a phase 2 trial of 84 virally suppressed, non-cirrhotic patients receiving stable NAs therapy, participants were assigned to various regimens of VIR-2218 (3 to 13 subcutaneous injections of 200 mg every 4 weeks) with or without PEG-IFNα. The highest rates of HBsAg loss were observed in longer-duration combination arms: 28% in cohort 4 (6 doses of VIR-2218 + 48 weeks PEG-IFNα) and 31% in cohort 5 (up to 13 doses of VIR-2218 + 44 weeks PEG-IFNα) at EOT, all sustained through 24 weeks off treatment. No HBsAg clearance occurred in monotherapy or short-duration groups. Among HBeAg-positive individuals, 46% achieved HBeAg loss or anti-HBe seroconversion [29]. Similarly, the REEF-1 study demonstrated that JNJ-3989 combined with NAs resulted in a mean HBsAg reduction of 2.6 log_10_. However, triple therapy (siRNA, CAM and NA) showed slightly less efficacy than dual therapy [30]. In the REEF-2 study (JNJ-3989 + JNJ-6379 + NA), after 48 weeks of treatment, HBV-DNA suppression was achieved, with a mean HBsAg reduction of 1.89 log_10_ IU/mL. Although functional cure was not achieved, more durable HBsAg reduction was observed, with some patients maintaining low HBsAg and minimal rebound up to 48 weeks off-treatment [31].

These findings highlight the potential of siRNA-based combinations, especially with PEG-IFNα, in virally suppressed, non-cirrhotic patients with low to moderate antigen burden. The dosing flexibility (e.g., monthly siRNA vs. weekly interferon) may enhance adherence and efficacy, particularly in patients aiming for finite-duration treatment and functional cure [29].

***Gene editing technology***

Gene-editing technologies offer a promising approach for CHB by directly targeting HBV cccDNA. Platforms such as ZFNs, TALENs, and CRISPR/Cas9 introduce double-stranded breaks (DSBs) in cccDNA, which are repaired via the host’s error-prone non-homologous end joining (NHEJ) pathway, resulting in indels that disrupt viral gene expression and replication [32,33].

Among gene-editing tools, CRISPR/Cas9 is particularly suited for HBV therapy due to its ease of retargeting via a 20-nt guide RNA, higher editing efficiency than ZFNs or TALENs, and compatibility with vectors like AAV or lipid nanoparticles [34]. Preclinical studies in cell lines, primary hepatocytes, and animal models demonstrated reductions in HBV DNA, HBsAg, and cccDNA, though durable HBsAg loss remains challenging and no human data are yet available [35].

Combining CRISPR/Cas9 with RNAi further enhanced antiviral effects—reducing HBsAg by 20–30% and cccDNA by up to 70% in vitro, and clearing HBsAg and HBcAg in mice [36]. Co-administration with ETV improved suppression of DHBV DNA (97–98%), though cccDNA clearance remained Cas9-dependent [37,38].

In vivo gene editing is also being clinically evaluated with PBGENE-HBV, an ARCUS®-based platform distinct from CRISPR, in the phase 1 ELIMINATE-B trial (NCT06680232) [39,40]. Further details are provided in Table S3.

These findings mark a significant advance, establishing a novel path toward HBV eradication by directly eliminating the viral reservoir. Going forward, efforts must focus on optimizing delivery, minimizing off-target effects, and refining combination regimens to translate pre-clinical potency into durable clinical success.

***Therapeutic vaccines***

Therapeutic vaccines are designed to overcome host immune tolerance and restore virus-specific immune responses against HBsAg. They function by activating HBV-specific T cells (including exhausted CD8⁺ T cells and T follicular helper cells) and reprogramming dysfunctional HBsAg-specific B cells, thereby promoting the generation of endogenous anti-HBs antibodies. These antibodies can neutralize circulating HBV virions and prevent viral entry into hepatocytes [41,42]. These vaccines vary in type, including protein-based, epitope peptide-based, and DNA-based formulations, and are generally applied after a reduction in viral load to amplify the antiviral immune response [42,43].

Protein-based vaccines such as VBI-2601 (BRII-179) are designed to induce both humoral and cellular immune responses. In clinical evaluations, VBI-2601 (BRII-179) elicited a robust antibody response, with more than 30% of participants developing anti-HBs antibodies; however, the decline in HBsAg levels remained modest [43,44]. The notable benefit of this vaccine is its capacity to induce anti-HBs antibodies, a feat difficult to achieve with monotherapy. Enhanced outcomes are observed when it is used in conjunction with siRNA drugs, such as BRII-835 (VIR-2218). A Phase Ib/IIa study of this combination therapy reported average HBsAg reductions of 1.7 to 1.8 log IU/mL, along with improved anti-HBs antibody and T-cell responses [45].

Epitope peptide-based vaccines are under early investigation and aim to present minimal epitopes to selectively activate HBV-specific T-cell responses. GS 4774 is a yeast-based, T cell–directed therapeutic vaccine expressing multiple HBV antigens (core, surface, and X proteins). In Phase II trials, GS 4774 monotherapy showed good safety and induced HBV-specific CD8⁺ T-cell responses, but had no significant impact on serum HBsAg levels in virally suppressed patients [46]. To enhance efficacy, GS 4774 was combined with tenofovir in a Phase II study, which showed increased production of IFN-γ, TNF, and IL-2 by CD8⁺ T cells, with minimal CD4⁺ T-cell activation. Although HBsAg levels remained unchanged, the vaccine’s strong CD8⁺ T-cell stimulation suggests potential value in combination strategies [47].

DNA-based vaccines aim to induce broad, durable T-cell responses against HBV antigens. A recent preclinical study by Maxime et al. (Hepatology, 2024) demonstrated in a murine model of chronic HBV that sequential administration of broadly neutralizing anti-HBs monoclonal antibodies followed by a DNA-based therapeutic vaccine markedly enhanced liver-infiltrating HBV-specific CD8⁺ T-cell and B-cell responses [48]. Although complete viral clearance was not achieved, the findings suggest that this combined immunotherapeutic approach can help reconstitute immune function in the setting of chronic infection. Building upon these findings, other therapeutic strategies have explored similar immune-restorative mechanisms. For instance, VTP-300, another therapeutic vaccine, prompts HBV-specific CD4⁺ and CD8⁺ T-cell responses and substantially lowers HBsAg levels, particularly in patients with initial HBsAg levels under 100 IU/mL [49]. A Phase II trial revealed that when VTP-300 was combined with nivolumab, a sustained HBsAg reduction was observed in some CHB patients, with 29% achieving a reduction of over 1 log after 169 days of follow-up [50]. Furthermore, a sequential treatment study with AB-729 (siRNA) followed by VTP-300 showed that after initial treatment with AB-729, 97% of patients (33/34) had HBsAg levels below 100 IU/mL [51]. Subsequent treatment with VTP-300 at 48 weeks ensured all participants maintained HBsAg levels below this threshold, compared to 75% in the placebo group.

Therapeutic vaccines are typically administered as part of a combination therapy regimen to patients who have already reduced their viral load through other treatments, with the goal of restoring immune functionality and achieving a functional cure. Integrating therapeutic vaccination with other immunotherapies and traditional antiviral treatments may enhance efficacy, either additively or synergistically [43]. They are particularly effective in patients with HBV viral loads below 10⁶ copies per mL, elevated ALT levels, or those who are HBeAg-negative [52].

***Monoclonal antibodies***

Monoclonal antibodies neutralize HBV particles, enhance antigen presentation by dendritic cells, and restore the function of HBV-specific CD8⁺ cytotoxic T lymphocytes (CTLs) by blocking immune checkpoints such as PD-1/PD-L1, thereby promoting clearance of infected hepatocytes [53].

VIR-3434 is a neutralizing antibody engineered to prevent HBV from binding to hepatocytes and to enhance immune system activation by lowering circulating HBsAg levels, thereby facilitating viral clearance [54]. In clinical trials with treatment-naïve patients, VIR-3434 exhibited substantial antiviral efficacy. The 75 mg and 300 mg single-dose cohorts saw decreases in HBsAg levels of −1.77 log and −1.83 log, respectively, alongside reductions in HBV DNA of −1.40 log and −2.03 log [55]. However, by the eighth week, a partial rebound in both HBsAg and HBV DNA levels was observed in both treatment-naïve and treatment-experienced groups.

Early outcomes from a Phase II trial that paired VIR-3434 with VIR-2218 (siRNA) indicated comparable rates of HBsAg loss during a 20- to 24-week treatment phase, regardless of whether it was used in conjunction with PEG-IFNα. At the end of treatment, 14.3% of participants in one group and 15% in another achieved HBsAg loss [28]. The combination was generally well tolerated, with most adverse events reported as mild.

These results underscore the potential of combining VIR-3434 with VIR-2218 as an effective strategy to enhance HBsAg clearance rates, presenting a promising new approach in therapy. Nonetheless, ongoing monitoring and additional research are required to confirm the long-term efficacy and safety of this therapeutic combination.

**Appendix S4. Challenges and future perspectives of combination therapy**

**Efficacy**

Achieving durable efficacy with new agents targeting HBsAg production remains a significant challenge, even in the combination with immunomodulators. Current data, such as those reported from Bepirovirsen or Xalnesiran, highlight the risk of relapse after therapy withdrawal, calling into question the suitability of functional cure as an early endpoint for these treatments. Functional cure in these studies remains limited in patients with baseline HBsAg levels higher than 1000 IU/mL—who are at the greater risk of liver-related complications. Future trials should explore combination of regimens with agents targeting different pathways to enhance response rates in these high-risk patients. Meanwhile, identifying patient subpopulations most likely to respond to specific treatments will be essential as well to optimize therapeutic outcomes and advance the efficacy of combination therapies.

**Drug safety and monitoring**

The safety and tolerability of novel therapeutic agents remain significant concerns, particularly when used in combination regimens. Most of these new drugs are still in the process of having their safety profiles more broadly validated through extended studies and wider clinical trials in different demographic settings. While combination therapies may improve antiviral efficacy, they also raise the risk of compounded or unforeseen side effects, which require careful evaluation. Long-term efficacy of the combination regimens will also need to be confirmed, particularly in patient subgroups presenting special conditions, such as liver cirrhosis and impaired immune states. Vigorous and continued monitoring of patients will be important for the correct forecasting of treatment responses, refining therapeutic approaches, and determining the timing for discontinuation of treatments. Post-marketing surveillance and real-world studies will be essential to capture delayed or rare adverse events. Biomarkers and predictive models to study combination therapies-associated risks will be significantly improved for detecting early signs of an adverse drug response. These are key components to heighten patient safety while providing additional effectiveness in treatment. Most importantly, the process would need a focused interest regarding personalized care for the patient, including comprehensive protocols in its monitoring [56].

**Drug resistance**

Resistance to drugs remains a formidable challenge in the treatment of CHB. There have been reports of resistance against first-line antiviral medications, such as ETV, thus showing that newer agents may face similar challenges [57]. While combination drugs with different mechanisms of action can reduce the chances of developing resistance compared with monotherapy, the HBV may still evolve resistance through alternative pathways. This requires close monitoring during the course of therapy for the detection of resistance-associated genetic mutations, with prompt adaptation in hospitals' treatment strategies. These may be the addition of new drug combinations or modification of current treatment regimens to newly emerging resistance mechanisms. With a better understanding of the biology behind HBV and technological development, more effective drugs will probably be developed, producing combinations that give higher functional cure rates with fewer side effects.

**Economic challenges**

The financial implications of applying combination therapies, especially in low- and middle-income countries (LMICs) where the burden of hepatitis B is highest,are enormous [58]. The high cost of multiple novel agents, limited health infrastructure, and lack of universal insurance coverage in these regions pose significant barriers to equitable access. Cost-effectiveness may improve through scaling up production, adopting international licensing strategies, and incorporating these therapies into global health programs supported by organizations such as the WHO [59]. Moreover, long-term benefits of a functional cure, such as lower healthcare costs related to cirrhosis and HCC, may provide the necessary justification for wider implementation of these therapies. However, without deliberate efforts to address pricing, distribution, and policy alignment, the potential public health impact of these therapies will remain limited in resource-constrained settings. These are the economic hurdles that must be overcome to achieve better access to innovative treatments and wider public health impact.

**Appendix S5 References**

[1] Nassal M. HBV cccDNA: viral persistence reservoir and key obstacle for a cure of chronic hepatitis B. Gut. 2015;64(12):1972–1984.

[2] Lucifora J, Protzer U. Attacking hepatitis B virus cccDNA—the holy grail to hepatitis B cure. J Hepatol. 2016;64:S41–S48.

[3] Tu T, Zhang H. Viral integrations in chronic hepatitis B infection: purposeless passenger or problematic promoter of persistence? Hepatology. 2022;76:15–17.

[4] Dandri M, Petersen J. cccDNA maintenance in chronic hepatitis B—targeting the matrix of viral replication. Infect Drug Resist. 2020;13:3873–3886.

[5] Terrault NA, Bzowej NH, Chang KM, Hwang JP, Jonas MM, Murad MH, et al. AASLD guidelines for treatment of chronic hepatitis B. Hepatology. 2016;63:261–283.

[6] Vaillant A. HBsAg, subviral particles, and their clearance in establishing a functional cure of chronic hepatitis B virus infection. ACS Infect Dis. 2021;7:1351–1368.

[7] D’Souza S, Lau KC, Coffin CS, Patel TR, et al. Molecular mechanisms of viral hepatitis–induced hepatocellular carcinoma. World J Gastroenterol. 2020;26:5759.

[8] Lu R, Zhang M, Liu ZH, Hao M, Tian Y, Li M, et al. Recurrence and influencing factors of hepatitis B surface antigen seroclearance induced by peginterferon alpha-based regimens. World J Gastroenterol. 2024;30:4725.

[9] Viganò M, Grossi G, Loglio A, Lampertico P, et al. Treatment of hepatitis B: is there still a role for interferon? Liver Int. 2018;38(Suppl 1):79–83.

[10] Bourlière M, Rabiega P, Ganne-Carrie N, Serfaty L, Marcellin P, Barthe Y, et al. Effect on HBs antigen clearance of addition of pegylated interferon alfa-2a to nucleos(t)ide analogue therapy versus nucleos(t)ide analogue therapy alone in patients with HBe antigen-negative chronic hepatitis B and sustained undetectable plasma hepatitis B virus DNA: a randomised, controlled, open-label trial. Lancet Gastroenterol Hepatol. 2017;2:177–188.

[11] Fanning GC, Zoulim F, Hou J, Bertoletti A, et al. Therapeutic strategies for hepatitis B virus infection: towards a cure. Nat Rev Drug Discov. 2019;18:827–844.

[12] Terrault NA, Lok AS, McMahon BJ, Chang KM, Hwang JP, Jonas MM, et al. Update on prevention, diagnosis, and treatment of chronic hepatitis B: AASLD 2018 hepatitis B guidance. Hepatology. 2018;67:1560–1599.

[13] Jindal A, Vyas AK, Kumar D, Kumar G, Sharma MK, Sarin SK, et al. Higher efficacy of pegylated interferon-α2b add-on therapy in hepatitis B envelope antigen-positive chronic hepatitis B patients on tenofovir monotherapy. Hepatol Res. 2018;48:451–458.

[14] Hoofnagle JH, Doo E, Liang TJ, Fleischer R, Lok AS, et al. Management of hepatitis B: summary of a clinical research workshop. Hepatology. 2007;45:1056–1075.

[15] Lok AS, Zoulim F, Dusheiko G, Chan HL, Buti M, Ghany MG, et al. Durability of hepatitis B surface antigen loss with nucleotide analogue and peginterferon therapy in patients with chronic hepatitis B. Hepatol Commun. 2020;4:8–20.

[16] Zhang H, Wang F, Zhu X, Chen Y, Chen H, Li X, et al. Antiviral activity and pharmacokinetics of the hepatitis B virus (HBV) capsid assembly modulator GLS4 in patients with chronic HBV infection. Clin Infect Dis. 2021;73(2):175–182.

[17] Zhang M, Gao Y, Kong F, Gao H, Yi Y, Wu C, et al. Efficacy and safety of GLS4 with entecavir vs entecavir alone in chronic hepatitis B patients: a multicenter clinical trial. J Infect. 2025;90(3):106446.

[18] Yuen MF, Asselah T, Jacobson IM, Brunetto MR, Janssen HL, Takehara T, et al. Efficacy and safety of the siRNA JNJ-73763989 and the capsid assembly modulator JNJ-56136379 (bersacapavir) with nucleos(t)ide analogues for the treatment of chronic hepatitis B virus infection (REEF-1): a multicenter, double-blind, active-controlled, randomized, phase 2b trial. Lancet Gastroenterol Hepatol. 2023;8(9):790–802.

[19] Nguyen L, Nguyen TT, Kim JY, Jeong JH. Advanced siRNA delivery in combating hepatitis B virus: mechanistic insights and recent updates. J Nanobiotechnology. 2024;22(1):745.

[20] Yuen MF, Schiefke I, Yoon JH, Ahn SH, Heo J, Kim JH, et al. RNA interference therapy with ARC-520 results in prolonged hepatitis B surface antigen response in patients with chronic hepatitis B infection. Hepatology. 2020;72(1):19–31.

[21] Hou J, Zhang W, Xie Q, Hua R, Tang H, Morano Amado LE, et al. Xalnesiran with or without an immunomodulator in chronic hepatitis B. N Engl J Med. 2024;391(22):2098–2109.

[22] Yuen MF, Locarnini S, Lim TH, Strasser SI, Sievert W, Cheng W, et al. Combination treatments including the small-interfering RNA JNJ-3989 induce rapid and sometimes prolonged viral responses in patients with CHB. J Hepatol. 2022;77(5):1287–1298.

[23] Yuen MF, Lim TH, Kim W, Tangkijvanich P, Yoon JH, Sievert W, et al. HBV RNAi inhibitor RG6346 in Phase 1b-2a trial was safe, well tolerated, and resulted in substantial and durable reductions in serum HBsAg levels. Paper presented at: The Liver Meeting Digital Experience™ 2020.

[24] Yuen R, Berliba E, Kim YJ, Holmes JA, Lim YS, Strasser SI, et al. Safety and pharmacodynamics of the GalNAc-siRNA AB-729 in subjects with chronic hepatitis B infection. Paper presented at: The Liver Meeting Digital Experience 2020.

[25] Yuen MF, Berliba E, Sukeepaisarnjaroen W, Tangkijvanich P, Leerapun A, Holmes J, et al. Low HBsAg levels maintained following cessation of the GalNAc-siRNA, AB-729, in chronic hepatitis B subjects on nucleos(t)ide analogue therapy. Paper presented at: Hepatology 2021.

[26] Gane E, Lim Y-S, Tangkijvanich P, O’Beirne J, Lim TH, Bakardjiev A, et al. Preliminary safety and antiviral activity of VIR-2218, an X-targeting HBV RNAi therapeutic, in chronic hepatitis B patients. J Hepatol. 2020. [https://doi.org/10.1016/S0168-8278(20)30647-4](https://doi.org/10.1016/S0168-8278(20)30647-4?utm_source=chatgpt.com)

[27] Gane EJ, Locarnini S, Lim TH, Strasser SI, Sievert W, Cheng W, et al. Dose response with the RNA interference (RNAi) therapy JNJ-3989 combined with nucleos(t)ide analogue (NA) treatment in expanded cohorts of patients with chronic hepatitis B (CHB). Paper presented at: The 70th Annual Meeting of the American Association for the Study of Liver Diseases (AASLD): The Liver Meeting 2019.

[28] Gane E, Jucov A, Dobryanska M, Yoon KT, Lim TH, Arizpe A, et al. Safety, tolerability, and antiviral activity of the siRNA VIR-2218 in combination with the investigational neutralizing monoclonal antibody VIR-3434 for the treatment of chronic hepatitis B virus infection: preliminary results from the phase 2 MARCH trial. Paper presented at: Hepatology 2022.

[29] Yuen MF, Lim YS, Yoon KT, Lim TH, Heo J, Tangkijvanich P, et al. VIR-2218 (elebsiran) plus pegylated interferon-alfa-2a in participants with chronic hepatitis B virus infection: a phase 2 study. Lancet Gastroenterol Hepatol. 2024;9(12):1121–1132.

[30] Yuen MF, Asselah T, Jacobson IM, Brunetto MR, Janssen HL, Takehara T, et al. Efficacy and safety of the siRNA JNJ-73763989 and the capsid assembly modulator JNJ-56136379 (bersacapavir) with nucleos(t)ide analogues for the treatment of chronic hepatitis B virus infection (REEF-1): a multicenter, double-blind, active-controlled, randomized, phase 2b trial. Lancet Gastroenterol Hepatol. 2023;8(9):790–802.

[31] Zhang H, Wang F, Zhu X, Chen Y, Chen H, Li X, et al. Antiviral activity and pharmacokinetics of the hepatitis B virus (HBV) capsid assembly modulator GLS4 in patients with chronic HBV infection. Clin Infect Dis. 2021;73(2):175–182.

[32] Martinez MG, Smekalova E, Combe E, Gregoire F, Zoulim F, Testoni B. Gene editing technologies to target HBV cccDNA. Viruses. 2022. [https://doi.org/10.3390/v14122654](https://doi.org/10.3390/v14122654?utm_source=chatgpt.com)

[33] Pacesa M, Pelea O, Jinek M. Past, present, and future of CRISPR genome editing technologies. Cell. 2024;187(5):1076–1100.

[34] Seeger C, Sohn JA. Complete spectrum of CRISPR/Cas9-induced mutations on HBV cccDNA. Mol Ther. 2016;24(7):1258–1266.

[35] Stone D, Long KR, Loprieno MA, Feelixge HS, Kenkel EJ, Liley RM, et al. CRISPR-Cas9 gene editing of hepatitis B virus in chronically infected humanized mice. Mol Ther Methods Clin Dev. 2021;20:258–275.

[36] Wang J, Chen R, Zhang R, Ding S, Zhang T, Yuan Q, et al. The gRNA-miRNA-gRNA ternary cassette combining CRISPR/Cas9 with RNAi strongly inhibits hepatitis B virus replication. Theranostics. 2017;7(12):3090–3105.

[37] Yao ZQ, Schank MB, Zhao J, El Gazzar M, Wang L, Zhang Y, et al. The potential of HBV cure: an overview of CRISPR-mediated HBV gene disruption. Front Genome Ed. 2024;6:1467449.

[38] Zheng Q, Bai L, Zheng S, Liu M, Zhang J, Wang T, et al. Efficient inhibition of duck hepatitis B virus DNA by the CRISPR/Cas9 system. Mol Med Rep. 2017;16(5):7199–7204.

[39] Gorsuch CL, Nemec P, Yu M, Xu S, Han D, Smith J, et al. Targeting the hepatitis B cccDNA with a sequence-specific ARCUS nuclease to eliminate hepatitis B virus in vivo. Mol Ther. 2022;30(9):2909–2922.

[40] Sulkowski M. Initial safety data from ELIMINATE-B, the first clinical trial of a gene editing treatment for chronic hepatitis B. EASL Congress 2025; May 7–10, 2025; Amsterdam, Netherlands.

[41] Hoogeveen RC, Boonstra A. Checkpoint inhibitors and therapeutic vaccines for the treatment of chronic HBV infection. Front Immunol. 2020;11:401.

[42] Zhang Y, Bourgine M, Wan Y, Song J, Li Z, Yu Y, et al. Therapeutic vaccination with lentiviral vector in HBV-persistent mice and two inactive HBsAg carriers. J Hepatol. 2024;80(1):31–40.

[43] Hoa PT, Huy NT, Le Thu T, Nga CN, Nakao K, Eguchi K, et al. Randomized controlled study investigating viral suppression and serological response following pre-S1/pre-S2/S vaccine therapy combined with lamivudine treatment in HBeAg-positive patients with chronic hepatitis B. Antimicrob Agents Chemother. 2009;53(12):5134–5140.

[44] Ma H, Lim TH, Leerapun A, Weltman M, Jia J, Lim YS, et al. Therapeutic vaccine BRII-179 restores HBV-specific immune responses in patients with chronic HBV in a phase Ib/IIa study. JHEP Rep. 2021;3(6):100361.

[45] Yuen MF, Wong G, Douglas M, Ma H, Zhu C, Ji Y, et al. Preliminary safety and efficacy of the combination therapy of BRII-835 (VIR-2218) and BRII-179 (VBI-2601) treating chronic HBV infection. Proceedings of the 32nd Annual Conference of the Asian Pacific Association for the Study of the Liver (APASL) 2023.

[46] Lok AS, Pan CQ, Han SH, Trinh HN, Fessel WJ, Rodell T, et al. Randomized phase II study of GS-4774 as a therapeutic vaccine in virally suppressed patients with chronic hepatitis B. J Hepatol. 2016;65(3):509–516.

[47] Boni C, Janssen HLA, Rossi M, Yoon SK, Vecchi A, Barili V, et al. Combined GS-4774 and tenofovir therapy can improve HBV-specific T-cell responses in patients with chronic hepatitis B. Gastroenterology. 2019;157(1):227–241.e227.

[48] Beretta M, Vesin B, Wei Y, Planchais C, Rosenbaum P, Ait-Goughoulte M, et al. Enhanced hepatitis B virus-specific immunity by combining neutralizing antibody therapy and DNA vaccination in a murine model of chronic hepatitis B virus infection. Hepatology. 2024. [https://doi.org/10.1097/HEP.0000000000001179](https://doi.org/10.1097/HEP.0000000000001179?utm_source=chatgpt.com)

[49] Tak WY, Chuang WL, Chen CY, Tseng KC, Lim YS, Lo GH, et al. Phase Ib/IIa randomized study of heterologous ChAdOx1-HBV/MVA-HBV therapeutic vaccination (VTP-300) as monotherapy and combined with low-dose nivolumab in virally suppressed patients with CHB. J Hepatol. 2024;81(6):949–959.

[50] Tait D, Bussey L, Kolenovska R, Downs M, Anderson K, Vardeu A, et al. VTP-300 immunotherapeutic, plus low-dose PD-1 inhibitor nivolumab, continues to show meaningful, sustained reductions in HBsAg levels. Proceedings of the European Association for the Study of the Liver (EASL) Annual Meeting; June 5–8, 2024; Milan, Italy.

[51] Agarwal K, Yuen MF, Roberts S, Lo GH, Hsu CW, Chuang WL, et al. OS-031 Imdusiran (AB-729) administered every 8 weeks for 24 weeks followed by the immunotherapeutic VTP-300 maintains lower HBV surface antigen levels in NA-suppressed CHB subjects than 24 weeks of imdusiran alone. J Hepatol. 2024;80:S26–S27.

[52] Michel ML, Deng Q, Mancini-Bourgine M. Therapeutic vaccines and immune-based therapies for the treatment of chronic hepatitis B: perspectives and challenges. J Hepatol. 2011;54:1286–1296.

[53] Beretta M, Mouquet H. Advances in human monoclonal antibody therapy for HBV infection. Curr Opin Virol. 2022;53:101205.

[54] Lempp FA, Volz T, Cameroni E, Benigni F, Zhou J, Rosen LE, et al. Potent broadly neutralizing antibody VIR-3434 controls hepatitis B and D virus infection and reduces HBsAg in humanized mice. J Hepatol. 2023;79:1129–1138.

[55] Agarwal K. A phase 1 study evaluating the neutralizing, vaccinal monoclonal antibody VIR-3434 in participants with chronic hepatitis B virus infection. Proceedings of the Hepatitis B: Novel Therapeutic Approaches (Viral Hepatitis); June 25, 2021; New York, NY, USA.

[56] Easterbrook PJ, Luhmann N, Bajis S, Min MS, Newman M, Lesi O, et al. WHO 2024 hepatitis B guidelines: an opportunity to transform care. Lancet Gastroenterol Hepatol. 2024;9(6):493–495.

[57] Hayashi S, Murakami S, Omagari K, Matsui T, Iio E, Isogawa M, et al. Characterization of novel entecavir resistance mutations. J Hepatol. 2015;63(3):546–553.

[58] World Health Organization (WHO). Global hepatitis report 2024: action for access in low- and middle-income countries. Geneva: World Health Organization; 2024. Available from: [https://www.who.int/publications/i/item/9789240091672](https://www.who.int/publications/i/item/9789240091672?utm_source=chatgpt.com). Accessed 27 June, 2025.

[59] Howell J, Pedrana A, Schroeder SE, Scott N, Aufegger L, Atun R, et al. A global investment framework for the elimination of hepatitis B. J Hepatol. 2021;74(3):535–549.
